# Molecular Design of Conjugated Small Molecule Nanoparticles for Synergistically Enhanced PTT/PDT

**DOI:** 10.1007/s40820-020-00474-6

**Published:** 2020-07-13

**Authors:** Wei Shao, Chuang Yang, Fangyuan Li, Jiahe Wu, Nan Wang, Qiang Ding, Jianqing Gao, Daishun Ling

**Affiliations:** 1 Institute of Pharmaceutics and Hangzhou Institute of Innovative Medicine, College of Pharmaceutical Sciences, Zhejiang University, Hangzhou, 310058 Zhejiang People’s Republic of China; 2 Jiangsu Breast Disease Center, The First Affiliated Hospital with Nanjing Medical University, Nanjing, 210029 Jiangsu People’s Republic of China

**Keywords:** Molecular design, Isoindigo, Conjugated small molecule nanoparticles, Singlet–triplet energy gap, Synergistic PTT/PDT

## Abstract

Simultaneous photothermal therapy (PTT) and photodynamic therapy (PDT) is beneficial for enhanced cancer therapy due to the synergistic effect. Conventional materials developed for synergistic PTT/PDT are generally multicomponent agents that need complicated preparation procedures and be activated by multiple laser sources. The emerging monocomponent diketopyrrolopyrrole (DPP)-based conjugated small molecular agents enable dual PTT/PDT under a single laser irradiation, but suffer from low singlet oxygen quantum yield, which severely restricts the therapeutic efficacy. Herein, we report acceptor-oriented molecular design of a donor–acceptor–donor (D–A–D) conjugated small molecule (IID-ThTPA)-based phototheranostic agent, with isoindigo (IID) as selective acceptor and triphenylamine (TPA) as donor. The strong D–A strength and narrow singlet–triplet energy gap endow IID-ThTPA nanoparticles (IID-ThTPA NPs) high mass extinction coefficient (18.2 L g^−1^ cm^−1^), competitive photothermal conversion efficiency (35.4%), and a dramatically enhanced singlet oxygen quantum yield (84.0%) comparing with previously reported monocomponent PTT/PDT agents. Such a high PTT/PDT performance of IID-ThTPA NPs achieved superior tumor cooperative eradicating capability in vitro and in vivo.

**Electronic supplementary material:**

The online version of this article (10.1007/s40820-020-00474-6) contains supplementary material, which is available to authorized users.

## Highlights

- A donor–acceptor–donor (D–A–D) conjugated small molecule IID-ThTPA with narrow singlet–triplet energy gap is synthesized *via* acceptor-oriented molecular design.
- IID-ThTPA nanoparticles exhibit not only competitive photothermal conversion efficiency (35.4%), but also a dramatically high singlet oxygen quantum yield (84.0%).
- IID-ThTPA nanoparticles enable superior cooperative tumor PTT/PDT eradicating capability both in vitro and in vivo.

**Electronic supplementary material:**

The online version of this article (10.1007/s40820-020-00474-6) contains supplementary material, which is available to authorized users.

## Graphical abstract

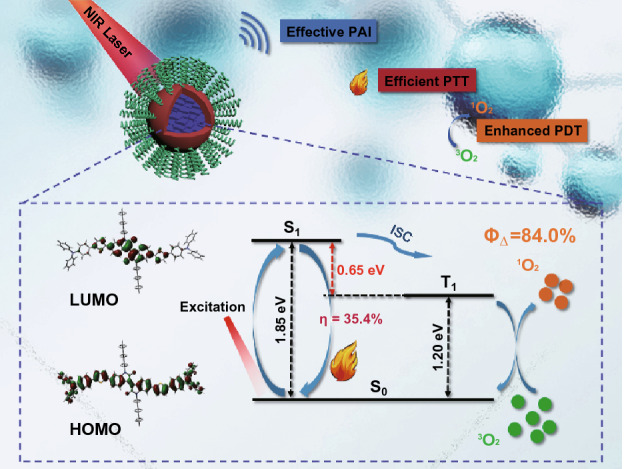

## Introduction

With high morbidity and mortality, cancer remains the leading cause of human death worldwide, and it is urgent to exploit efficient theranostics for cancer [1]. As emerging phototherapeutic modalities, photothermal therapy (PTT) and photodynamic therapy (PDT) have received increasing attention for cancer therapy in recent years [2–4]. In contrast to conventional therapies such as surgery, radiotherapy, and chemotherapy, phototherapy has several advantages, including remote controllability, low side effects, and noninvasiveness [5]. According to Jablonski diagram, upon near-infrared (NIR) laser irradiation, photosensitizer (PS) in ground state (S_0_) is excited to its excited states (S_*n*_) with higher energy. Based on Kasha’s rule, the excited states are unstable and subsequently undergo nonradiative relaxation called internal conversion (IC), to the lowest excited state (S_1_) [6]. The excited PS in S_1_ state can relax to ground state via nonradiative relaxation with heat energy release, which is the basic mechanism of PTT [6]. Alternatively, for PDT, excited PS in S_1_ state undergoes singlet-to-triplet intersystem crossing (ISC) to the triplet states (T_*n*_), followed by energy transfer from the lowest excited triplet state (T_1_) to the surrounding molecular oxygen (^3^O_2_) to generate a kind of reactive oxygen species (ROS), cytotoxic singlet oxygen (^1^O_2_), to kill cancer cells [6]. Recently, integrating PTT and PDT in a sole system has been successful in synergistic cancer therapy. By combining PTT and PDT, the PDT efficacy can be photothermally enhanced, wherein the photothermal effect can accelerate intratumoral blood flow so as to increase the oxygen supplement in tumor to amplify the PDT efficacy [7, 8].

Despite the satisfactory therapeutic outcome, currently available PTT/PDT agents are mainly multicomponent materials [9, 10]. Due to the intrinsic structural characteristics, these agents are generally prepared by sophisticated procedures and must be excited by different light sources for PTT and PDT, thus leading to the stability issue and operational complexity [11–14]. To overcome these shortcomings, it is of great significance to explore efficient monocomponent-based PTT/PDT agents that can be excited by a single laser source.

**Scheme 1:**
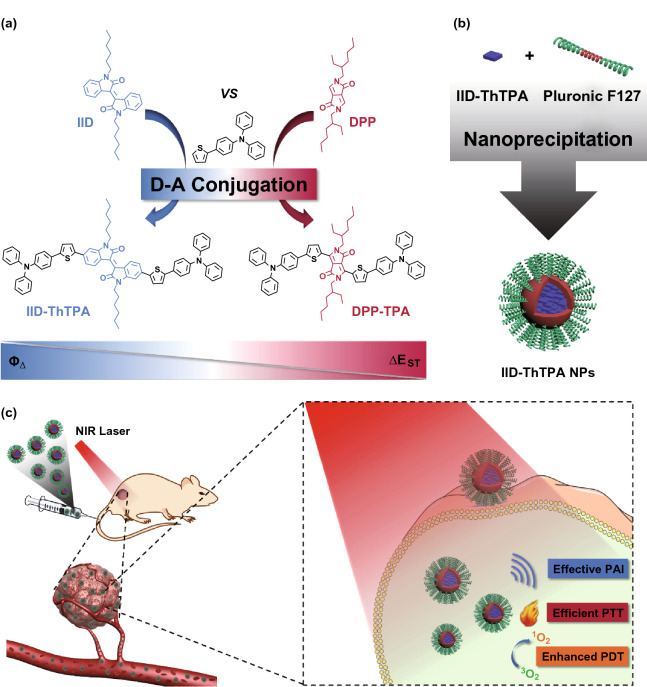
Illustration of molecular design of IID-ThTPA, preparation of IID-ThTPA NPs, and biomedical application of IID-ThTPA NPs. **a** Acceptor-oriented molecular design of IID-ThTPA with narrow singlet–triplet energy gap. **b** IID-ThTPA NPs prepared by a nanoprecipitation method. **c** PAI-guided synergistically enhanced PTT/PDT under a single NIR laser irradiation enabled by IID-ThTPA NPs

Donor–acceptor (D–A) conjugated small molecules (CSMs) with excellent chemical and photothermal stability and fascinating optical property have been widely used in organic optoelectronics [15], chemical sensing [16], and bioimaging [17–19]. With the advantages of chemically defined structures, high purity, good reproducibility, facile modification, and easy processability, CSMs have shown great potential in biomedical applications [2]. Upon NIR laser excitation, the specific photophysical processes of CSMs can be used to perform PTT (nonradiative relaxation from S_1_ to S_0_ to generate heat) and PDT (ISC from S_1_ to T_1_, followed by energy transfer from T_1_ to surrounding ^3^O_2_ to generate ^1^O_2_) simultaneously. Based on the various kinds of acceptors [20], a plenty of CSMs and conjugated polymers (CPs) have been developed for PTT and PDT [21–28]. Diketopyrrolopyrrole (DPP) is the most popular acceptor being used to construct CSMs for synergistic PTT/PDT under a single NIR laser irradiation [21–23, 26]. However, most of these agents suffer from low ^1^O_2_ quantum yield (*Φ*_Δ_), which severely restricts the therapeutic efficacy. The low *Φ*_Δ_ originates from the inefficient ISC of the PSs, and a general method to overcome this shortcoming is the introduction of heavy atoms (e.g., Br, I, Te, Ru, Ir, and Hf) to induce the “heavy atom effect” for enhanced ISC [29–34]. However, such a methodology often causes concerns about the increased “dark toxicity” and cost [35–37]. Therefore, efforts have been devoted to explore heavy atom-free PSs with high *Φ*_Δ_ [38], but the development still falls behind. Based on these considerations, it is highly urgent to exploit advanced pure organic CSMs with both high photothermal conversion efficiency (*η*) and *Φ*_Δ_. Herein, we report acceptor-oriented molecular design of a novel D–A–D CSM (IID-ThTPA) with isoindigo (IID) as selective acceptor and triphenylamine (TPA) as donor for synergistically enhanced PTT/PDT. IID, a naturally occurring isomer of indigo, is a representative acceptor with good backbone coplanarity [39–42]. Compared with the previously reported DPP-based nanoparticles, the nanoparticles of IID-ThTPA (IID-ThTPA NPs) exhibit comparable photothermal conversion efficiency (35.4%), and a much enhanced *Φ*_Δ_ (84.0%) contributed by the narrow singlet–triplet energy gap (ΔE_ST_) of IID-ThTPA as validated by density functional theory (DFT) calculation. In vitro results show minimal dark cytotoxicity of IID-ThTPA NPs against 4T1 cells, and PTT or PDT can be performed individually under 671-nm laser irradiation. Importantly, synergistic PTT/PDT exhibits superior phototherapeutic effect to PTT or PDT alone. With good biocompatibility and colloidal stability, synergistic PTT/PDT with improved tumor-eradicating capability is further achieved in vivo under the guidance of photoacoustic imaging (PAI) (Scheme 1). Considering the diversity of D and A units, one can adjust the energy level of the excited states of the CSMs by combining different D and A units. Consequently, the specific photophysical processes related to PTT (and/or PDT) can be manipulated and various efficient phototheranostic agents may be discovered.

## Results and Discussion

### Synthesis of IID-ThTPA and Preparation of IID-ThTPA NPs

**Fig. 1:**
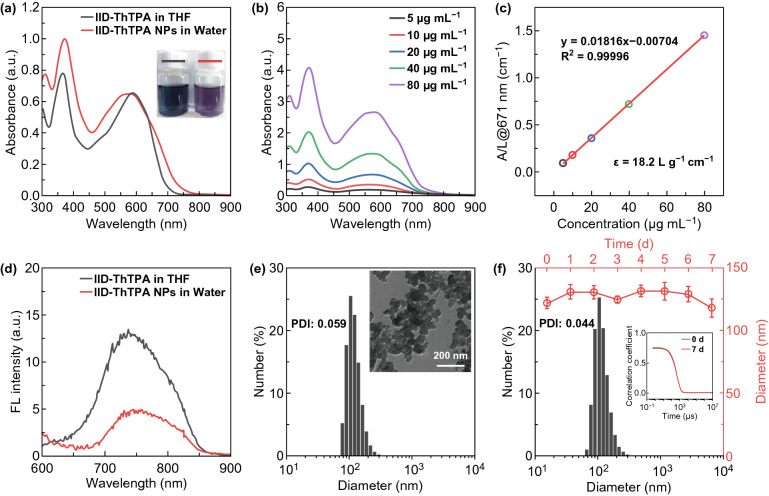
Basic properties of IID-ThTPA and IID-ThTPA NPs. **a** UV–Vis–NIR absorption spectra of IID-ThTPA in THF and IID-ThTPA NPs in water (20 μg mL^−1^) (insets are the digital photographs of IID-ThTPA in THF and IID-ThTPA NPs in water). **b** UV–Vis–NIR absorption spectra of IID-ThTPA NPs with different concentrations. **c** Mass extinction coefficient of IID-ThTPA NPs at 671 nm. Normalized absorbance intensity at 671 nm divided by the characteristic length of the cell (A/L) at different concentrations (mass extinction coefficient is calculated by the Lambert–Beer law: *A*/*L* = *ε*c (L = 1 cm)). **d** Fluorescence spectra of IID-ThTPA in THF and IID-ThTPA NPs in water (20 μg mL^−1^) (excitation wavelength: 550 nm). **e** DLS profile of freshly prepared IID-ThTPA NPs (inset shows the TEM image of IID-ThTPA NPs). **f** DLS profile of IID-ThTPA NPs after one week’s storage at room temperature and the size change of IID-ThTPA NPs during one week’s storage at room temperature (inset is the autocorrelation functions of IID-ThTPA NPs at 0 and 7 days, respectively)

IID-ThTPA was synthesized by Stille coupling reaction between IID-Br and Sn-ThTPA in a yield of 70% (Scheme S1). The chemical structures of key intermediates and final product were characterized by ^1^H, ^13^C nuclear magnetic resonance (NMR) spectra and high-resolution matrix-assisted laser desorption/ionization time-of-flight (MALDI-TOF) mass spectrum (Figs. S1–S5). Two thiophene (Th) rings are inserted into the backbone of IID-ThTPA between D and A units acting as π-bridges to promote intramolecular charge transfer (ICT) for high absorption coefficient in NIR. Two hexyls are attached to the IID segment to ensure the organic solvent processability of IID-ThTPA. IID-ThTPA can dissolve easily in common organic solvents, such as tetrahydrofuran (THF), dichloromethane, and chloroform. In THF, IID-ThTPA exhibits two main absorption peaks at 365 and 588 nm with absorption onset extending to 700 nm (Fig. 1a). The former peak is ascribed to the local π–π* transition of the conjugated backbone, while the latter one originates from the ICT transition between D and A units. The mass extinction coefficient (*ε*) of IID-ThTPA at 588 nm is as high as 33.2 L g^−1^ cm^−1^ (Fig. S6).

To endow water solubility of IID-ThTPA for further biomedical application, IID-ThTPA-based nanoparticles (IID-ThTPA NPs) were prepared by a nanoprecipitation method using Pluronic F127 as encapsulating matrix. The absorption spectrum profile of IID-ThTPA NPs is similar to IID-ThTPA in THF, but broadening to some extent. It is worth noting that a shoulder absorption at ~ 667 nm appears in the absorption spectrum of IID-ThTPA NPs (Fig. 1a). These evidences suggest much stronger intermolecular interactions in IID-ThTPA NPs as compared to molecular IID-ThTPA in solution. The loading efficiency of IID-ThTPA in IID-ThTPA NPs was calculated to be 73% by an ultraviolet–visible–near-infrared (UV–Vis–NIR) spectroscopic method (Fig. S7). IID-ThTPA NPs have a high mass extinction coefficient of 18.2 L g^−1^ cm^−1^ at the NIR laser excitation wavelength of 671 nm, revealing good light-absorbing capability (Fig. 1b, c). Moreover, as a result of aggregation-caused quenching (ACQ) effect, the fluorescence intensity of IID-ThTPA NPs in water decreases to 37% of IID-ThTPA in THF and the quenched radiative energy may contribute to the photothermal conversion of IID-ThTPA NPs (Fig. 1d). The morphology of IID-ThTPA NPs was characterized by transmission electron microscopy (TEM) as dispersed spherical nanoparticles with size of ~ 60 nm. Dynamic light scattering (DLS) measurement suggests the hydrodynamic diameter of IID-ThTPA NPs is ~ 120 nm with a polydispersed index (PDI) of 0.059 (Fig. 1e). The appropriate size of the as-prepared IID-ThTPA NPs is suitable for tumor accumulation due to the enhanced penetration and retention (EPR) effect [43]. There are no noticeable size changes in IID-ThTPA NPs during one week’s storage in various mediums (Figs. 1f and S8), indicating their excellent colloidal stability for biomedical application.

### Photothermal Effect of IID-ThTPA NPs

**Fig. 2:**
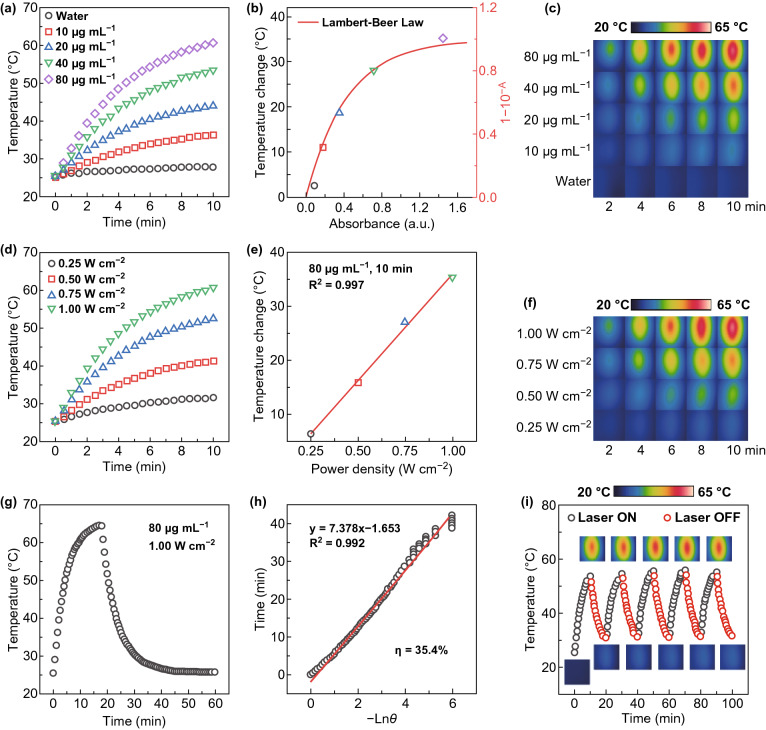
Photothermal effect of IID-ThTPA NPs. **a** Photothermal heating curves of IID-ThTPA NPs with different concentrations (671 nm, 1.00 W cm^−2^). **b** Plot of the temperature changes of IID-ThTPA NPs versus the absorbance at different concentrations with a fitted curve derived from Lambert–Beer law. **c** Infrared thermographs of IID-ThTPA NPs with different concentrations under laser irradiation at various time points (671 nm, 1.00 W cm^−2^). **d** Photothermal heating curves of IID-ThTPA NPs (80 μg mL^−1^) under 671-nm laser irradiation with different power densities. **e** Plot of the temperature changes of IID-ThTPA NPs versus the laser power densities with a linearly fitted line. **f** Infrared thermographs of IID-ThTPA NPs (80 μg mL^−1^) under 671-nm laser irradiation with different power densities at various time points. **g** Photothermal heating curve to calculate the photothermal conversion efficiency. IID-ThTPA NPs (80 μg mL^−1^) were irradiated by a 671-nm laser with a power density of 1.00 W cm^−2^ to reach a temperature plateau; then, the laser was shut off and the temperature was allowed to cool to room temperature naturally. **h** Plot of linear time data from the cooling period of IID-ThTPA NPs versus negative natural logarithm of the driving force temperature (*θ*). **i** Photothermal heating curve of IID-ThTPA NPs (40 μg mL^−1^) for five laser on/off cycles under irradiation (671 nm, 1.00 W cm^−2^) (insets are the infrared thermographs of IID-ThTPA NPs at different time points during the laser on/off cycles)

The intense NIR absorption of IID-ThTPA NPs encouraged us to explore their photothermal effect. Under laser irradiation (671 nm, 1.00 W cm^−2^, 10 min), IID-ThTPA NPs with different concentrations all show temperature elevation, and the final temperatures after laser irradiation are 27.7, 36.3, 43.9, 53.4, and 60.5 °C for IID-ThTPA NPs with concentrations of 0, 10, 20, 40, and 80 μg mL^−1^, respectively (initial temperature: 25 °C) (Fig. 2a). Plot of the temperature changes of IID-ThTPA NPs versus the absorbance at different concentrations can be fitted by a curve derived from Lambert–Beer law, which implies the well utilization of the absorbed light energy (Fig. 2b). The temperature elevation of IID-ThTPA NPs during laser irradiation is also visualized by infrared thermographs (Fig. 2c). In addition, photothermal effects under different laser power densities were also investigated. A higher laser power density leads to a faster temperature elevation (Fig. 2d), and plot of the temperature changes of IID-ThTPA NPs versus the laser power densities can be linearly fitted with *R*^2^ = 0.997 (Fig. 2e). The photothermal effects under different laser power densities are also validated by infrared thermographs (Fig. 2f).

In order to calculate the photothermal conversion efficiency, IID-ThTPA NPs solution (80 μg mL^−1^) was subjected to laser irradiation (671 nm, 1.00 W cm^−2^) to reach a temperature plateau, then the laser was shut off to allow it cool to room temperature naturally (Fig. 2g). By plotting the linear time data from the cooling period of IID-ThTPA NPs versus negative natural logarithm of the driving force temperature, the time constant (*τ*) of the system can be determined to be 7.378 min (Fig. 2h), which is used to calculate the photothermal conversion efficiency of 35.4% based on a method reported by Roper *et al*. [44]. The high photothermal conversion performance of IID-ThTPA NPs can be maintained even after five laser on/off cycles, suggesting the excellent photothermal stability (Fig. 2i). Moreover, the photostability of IID-ThTPA NPs was also compared with a clinically used photosensitizer indocyanine green (ICG). The absorption spectrum of ICG decreases with time under laser irradiation, while that of IID-ThTPA NPs keeps constant (Fig. S9). All these results suggest IID-ThTPA NPs can be used as an efficient PTT agent with high photostability.

### Singlet Oxygen-Generating Capability of IID-ThTPA NPs

**Fig. 3:**
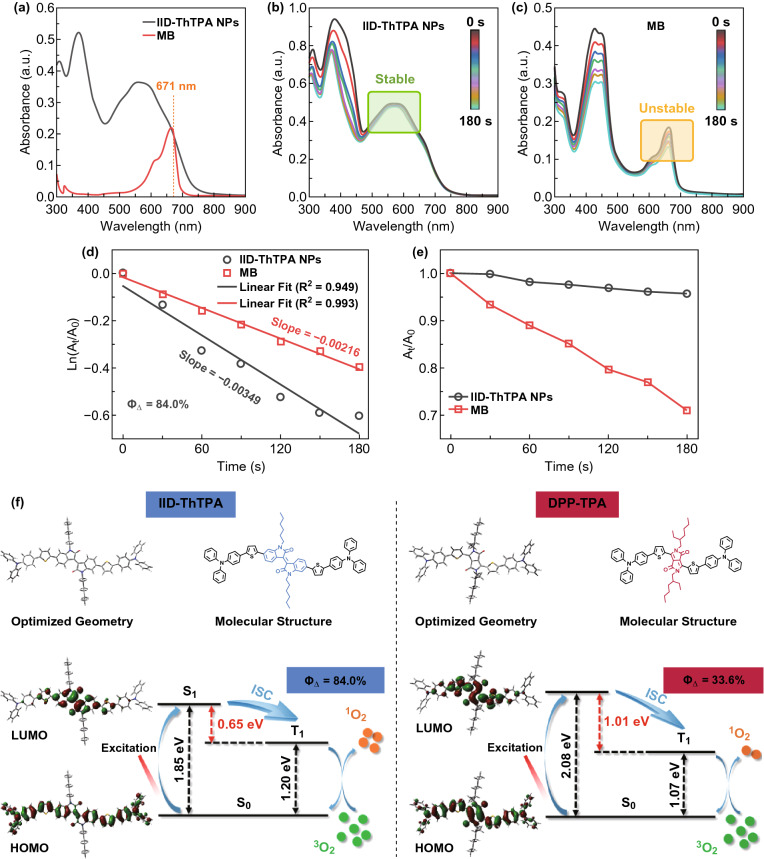
Singlet oxygen-generating capability of IID-ThTPA NPs. **a** UV–Vis–NIR absorption spectra of IID-ThTPA NPs and a standard reference MB in water. Degradation of DPBF in the presence of **b** IID-ThTPA NPs and **c** MB under laser irradiation. Degradation kinetics of DPBF in the presence of IID-ThTPA NPs and MB under laser irradiation, where **d** A_0_ and A_t_ are the characteristic absorbance of DPBF at 410 nm before and after laser irradiation, respectively; **e** A_0_ and A_t_ are the absorbance at 580 nm (IID-ThTPA NPs) or 665 nm (MB) before and after laser irradiation, respectively. **f** DFT-calculated optimized geometries, HOMOs, LUMOs, S_1_, and T_1_ energy levels of IID-ThTPA and a previously reported conjugated small molecule DPP-TPA to depict the ultrahigh singlet oxygen quantum yield of IID-ThTPA NPs

To identify the type of generated ROS in our system, electron spin resonance (ESR) measurement was taken using 2,2,6,6-tetramethylpiperidine (TEMP) as the ^1^O_2_ indicator and 5-*tert*-butoxycarbonyl-5-methyl-1-pyrroline-*N*-oxide (BMPO) as the hydroxyl radical and superoxide anion radical indicator. Under laser irradiation, TEMP exhibits a characteristic ^1^O_2_-induced signal in the presence of IID-ThTPA NPs, which increases with laser irradiation time (Fig. S10a). In contrary, no other ROS such as hydroxyl radical and superoxide anion radical can be detected using BMPO (Fig. S10b). These results indicate the type of ROS in our system is ^1^O_2_. We further investigated the singlet oxygen-generating capability of IID-ThTPA NPs by using 1,3-diphenylisobenzofuran (DPBF) as a singlet oxygen probe. The absorption spectra of IID-ThTPA NPs and methylene blue (MB) water solutions were measured, and the absorbance of these two samples at the laser excitation wavelength of 671 nm was adjusted to a same value of ~ 0.2 (Fig. 3a). Under a 671-nm laser irradiation, the characteristic absorbance of DPBF at 410 nm decreases rapidly with time in the presence of IID-ThTPA NPs due to the ^1^O_2_-induced oxidation of DPBF, indicating a remarkable singlet oxygen-generating capability of IID-ThTPA NPs. As a standard reference (*Φ*_Δ_ = 52.0%), MB solution mixed with DPBF was subjected to the same experimental condition, but the characteristic absorbance of DPBF decreases much slower than that of IID-ThTPA NPs (Fig. 3b, c). According to the DPBF degradation dynamics curves under laser irradiation in the presence of IID-ThTPA NPs and MB, the singlet oxygen quantum yield of IID-ThTPA NPs was calculated to be 84.0% (Fig. 3d), which, to our knowledge, is highly competitive among the single laser-excited dual PTT/PDT monocomponent agents reported thus far (Table S1) [21–23, 26, 45–51]. A better photostability of IID-ThTPA NPs against MB is also demonstrated by monitoring the degradation of IID-ThTPA NPs (absorbance at 580 nm) and MB (absorbance at 665 nm) under laser irradiation (Fig. 3e). In addition, we investigated the ^1^O_2_-generating capability of IID-ThTPA NPs using singlet oxygen sensor green (SOSG). Under laser irradiation, an obvious time-dependent fluorescence increment of SOSG at 532 nm can be observed in the presence of IID-ThTPA NPs, while there is no such effect in the absence of IID-ThTPA NPs, further demonstrating the robust ^1^O_2_-generating capability of IID-ThTPA NPs (Fig. S11).

For a deeper insight into the ultrahigh singlet oxygen quantum yield of IID-ThTPA NPs, density functional theory (DFT) calculation was performed for IID-ThTPA to study its optimized geometry, frontier orbital distributions, S_1_, and T_1_ energy levels. A reported conjugated small molecule DPP-TPA (*Φ*_Δ_ = 33.6%, DPP-TPA NPs) based on a widely used acceptor of DPP was also calculated for comparison. Both IID-ThTPA and DPP-TPA possess a rigid and coplanar molecular backbone, and both the highest occupied molecular orbitals (HOMOs) of IID-ThTPA and DPP-TPA distribute along the whole conjugated backbones, while the lowest unoccupied molecular orbitals (LUMOs) mainly locate on the acceptors of IID-ThTPA and DPP-TPA. However, the much larger HOMO–LUMO separation and twisted conformation of IID-ThTPA lead to a narrow Δ*E*_ST_ of 0.65 eV, which is almost half of that of DPP-TPA (1.01 eV). The narrow Δ*E*_ST_ of IID-ThTPA can facilitate the ISC from S_1_ to T_1_ to increase the T_1_ population, thus generating much more singlet oxygen than DPP-TPA NPs (Fig. 3f, Table S2), which is consistent with a recent work [52]. These results along with the results in previous section make IID-ThTPA NPs an efficient PTT/PDT agent.

### In Vitro Phototherapy

**Fig. 4:**
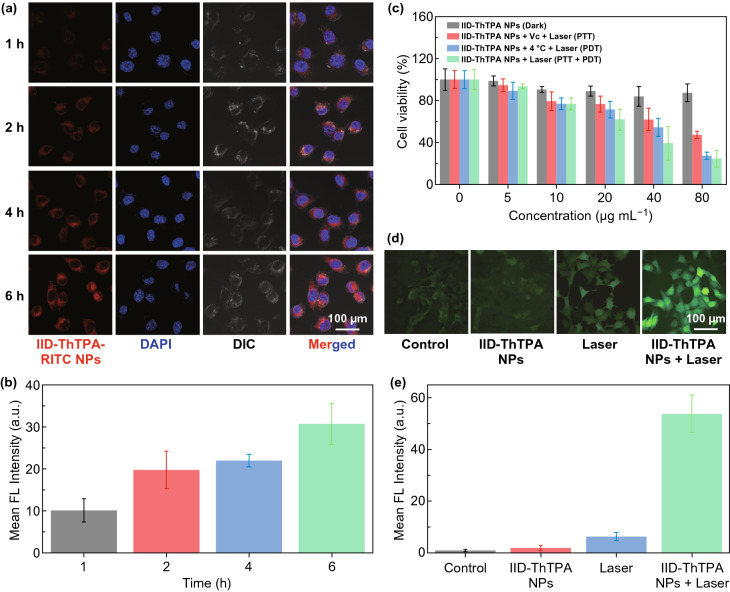
In vitro phototherapy of IID-ThTPA NPs. **a** CLSM images of 4T1 cells after incubating with IID-ThTPA-RITC NPs for different times (1, 2, 4, and 6 h). **b** Quantitative analysis of RITC fluorescence intensity in **a**. **c** Cell viability of 4T1 cells incubating with IID-ThTPA NPs (0, 5, 10, 20, 40, and 80 μg mL^−1^) under different experimental conditions (dark, PTT, PDT, and PTT + PDT) (the results are presented as mean ± SD, *n* = 6). **d** Intracellular ROS level of 4T1 cells under different treatments (control, IID-ThTPA NPs, laser, and IID-ThTPA NPs + laser). **e** Quantitative analysis of DCF fluorescence intensity in **d**

The cellular uptake of IID-ThTPA NPs was evaluated before in vitro phototherapy, rhodamine isothiocyanate (RITC) was coprecipitated with IID-ThTPA to prepare fluorescent IID-ThTPA-RITC NPs. Confocal laser scanning microscope (CLSM) images clearly show a time-dependent cellular uptake behavior of IID-ThTPA-RITC NPs (Fig. 4a, b). IID-ThTPA NPs exhibit negligible dark cytotoxicity (cell viability > 80%) toward 4T1 cells even at a high concentration of 80 μg mL^−1^. To perform PTT only, 4T1 cells were coincubated with a ROS scavenger Vitamin C (Vc) and then subjected to laser irradiation (671 nm, 1.00 W cm^−2^, 5 min). The cell viability decreases with increased IID-ThTPA NPs concentration, and the IC_50_ value under this condition is determined to be ~ 73.2 μg mL^−1^. For PDT only, 4T1 cells were kept at 4 °C during laser irradiation (671 nm, 1.00 W cm^−2^, 5 min) to avoid temperature elevation. An IID-ThTPA NPs concentration-dependent cancer cell-killing effect is also observed, and the IC_50_ value is ~ 46.6 μg mL^−1^. These results suggest IID-ThTPA NPs can perform both PTT and PDT, while the PDT effect toward 4T1 cells is stronger than PTT effect, given the ultrahigh *Φ*_Δ_ of IID-ThTPA NPs. Finally, we conducted synergistic PTT/PDT toward 4T1 cells by direct laser irradiation. A much enhanced therapeutic effect with a IC_50_ value of ~ 30.6 μg mL^−1^ is observed due to the synergistic effect of PTT and PDT (Fig. 4c). Furthermore, we investigated the intracellular ROS level of 4T1 cells under different treatments using nonfluorescent 2’,7’-dichlorofluorescein diacetate (DCFH-DA) as a probe, which can be rapidly converted into green fluorescent 2’,7’-dichlorofluorescein (DCF) by ROS. There is a significant ROS generation of the cells treated with IID-ThTPA NPs under laser irradiation, in sharp contrast with those in control group that merely show negligible ROS generation (Fig. 4d, e).

### In Vivo PAI-Guided Phototherapy

**Fig. 5:**
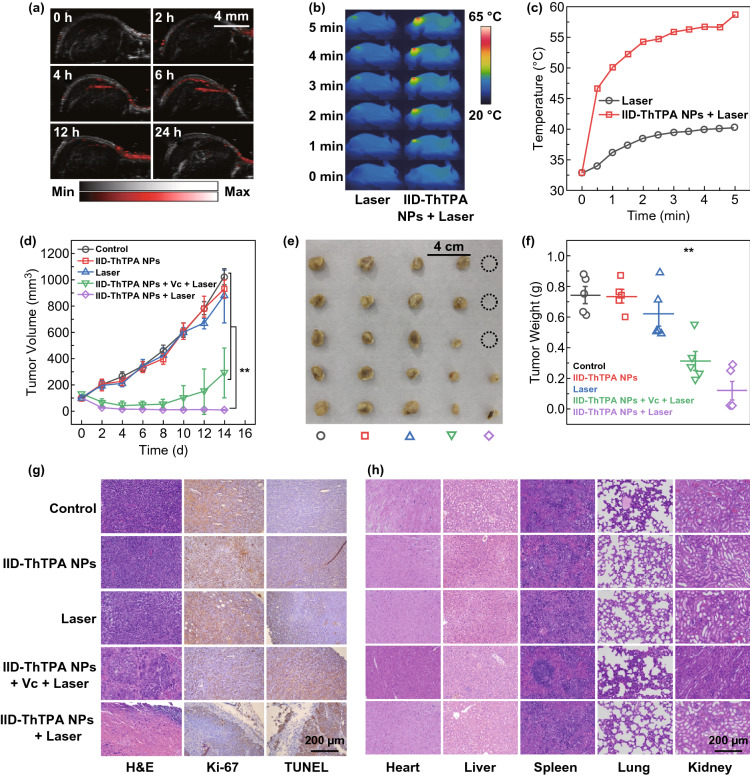
In vivo PAI-guided phototherapy enabled by IID-ThTPA NPs. **a** PA images of the tumor sites at 680 nm at different time points post-injection (0, 2, 4, 6, 12, and 24 h). **b** Infrared thermographs of the mice under laser irradiation (671 nm, 1.00 W cm^−2^) in laser and IID-ThTPA NPs + laser groups. **c** Tumor temperature elevation curves of the mice under laser irradiation (671 nm, 1.00 W cm^−2^) in laser and IID-ThTPA NPs + laser groups. **d** Tumor volume variation curves of the mice in control, IID-ThTPA NPs, laser, IID-ThTPA NPs + Vc + laser (PTT), and IID-ThTPA NPs + laser (PTT + PDT) groups during the treatment period (the results are presented as mean ± SD, **P < 0.01 by two-tailed unpaired Student’s t tests, *n* = 5). **e** Digital photographs of the tumors dissected from the mice in control, IID-ThTPA NPs, laser, IID-ThTPA NPs + Vc + laser (PTT), and IID-ThTPA NPs + laser (PTT + PDT) groups at the end of treatment. **f** Tumor weights of the mice in control, IID-ThTPA NPs, laser, IID-ThTPA NPs + Vc + laser (PTT), and IID-ThTPA NPs + laser (PTT + PDT) groups at the end of treatment (the results are presented as mean ± SD, **P < 0.01 by two-tailed unpaired Student’s t tests, *n* = 5). **g** H&E, Ki-67, and TUNEL staining of tumors of the mice after different treatments. **h** H&E staining of the major organs (heart, liver, spleen, lung, and kidney) of the mice after different treatments

Prior to in vivo study, we measured the PA spectra of IID-ThTPA NPs. The PA signal, which originates from the photothermia-induced elastic expansion of the medium, is directly related to the light absorption of IID-ThTPA NPs (Fig. S12a), and the PA intensities of IID-ThTPA NPs at 680 nm can be linearly plotted versus their concentrations (*R*^2^ = 0.9942); consequently, the PAI can readily be used to quantify the localized concentration of IID-ThTPA NPs in tumor (Fig. S12b). After intravenous injection of IID-ThTPA NPs (200 μL, 800 μg mL^−1^ based on IID-ThTPA) into orthotopic 4T1 tumor-bearing mice, the PA signal intensity in tumor site gradually increases with time and reaches its maximum at 6 h post-injection (Fig. 5a and S12c), which indicates the maximum tumor accumulation of IID-ThTPA NPs; thus, the time point for laser irradiation in the in vivo phototherapy was set to be 6 h post-injection. The pharmacokinetic profile of IID-ThTPA NPs was fitted by a two-compartment model, and the blood circulation half-life was calculated to be ~ 2.2 h (Fig. S13). To evaluate the in vivo phototherapeutic efficacy of IID-ThTPA NPs, the tumor-bearing mice were randomly divided into five groups (*n* = 5): control (without any treatment), IID-ThTPA NPs (only treated with IID-ThTPA NPs), laser (only treated with laser), IID-ThTPA NPs + Vc + laser (PTT) (treated with IID-ThTPA NPs, Vc, and laser), and IID-ThTPA NPs + laser (PTT + PDT) (treated with IID-ThTPA NPs and laser). Under laser irradiation (671 nm, 1.00 W cm^−2^, 5 min), the temperature of tumor site of the mice in laser group only reaches 40.2 °C, while the temperature of tumor site of the mice in IID-ThTPA NPs + laser group increases rapidly to 58.7 °C, which is sufficient for tumor ablation (Fig. 5b, c). The tumor volume changes were monitored every 2 days. In the groups of control, IID-ThTPA NPs, and laser, the tumors grow rapidly from an initial volume of ~ 100 mm^3^ to 800–1100 mm^3^ in 2 weeks. Although the tumors of the mice in IID-ThTPA NPs + Vc + laser (PTT) group are inhibited in the first 6 days, an obvious tumor regrowth is observed in the next 8 days. Dramatically, the tumors of the mice in IID-ThTPA NPs + laser (PTT + PDT) group shrink continuously during the treatment period and nearly disappear at the end of treatment (Figs. 5d–f and S14), and the in vivo tumor phototherapy is also verified by H&E, Ki-67, and TUNEL staining of the tumors in different groups (Fig. 5g), suggesting the best phototherapeutic effect of IID-ThTPA NPs + laser (PTT + PDT) group. Positive staining of the Ki-67, known as a proliferation marker, shows the lowest expression (~ 10%) in tumors in IID-ThTPA NPs + laser (PTT + PDT) group compared to the control group (~ 72%) (Fig. S15a). Quantitative analysis of TUNEL staining in Fig. 5g also shows a similar result: The apoptosis rate of tumor cells in IID-ThTPA NPs + laser (PTT + PDT) group is 17.8-fold higher than the control group (Fig. S15b).

**Fig. 6:**
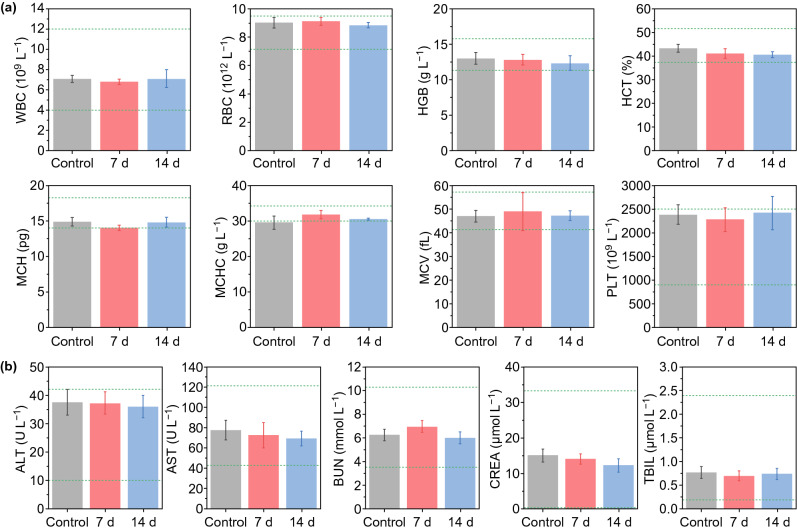
In vivo toxicity evaluation. **a** Hematological index (WBC, RBC, HGB, HCT, MCH, MCHC, MCV, and PLT) of the mice in control, 7 d post-injection, and 14 d post-injection groups. **b** Biochemical blood analysis (ALT, AST, BUN, CREA, and TBIL) of the mice in control, 7 d post-injection, and 14 d post-injection groups (the results are presented as mean ± SD, *n* = 3)

In addition, the biodistribution of the nanoparticles was investigated, and negligible fluorescence signals in major organs could be detected on the 3rd day after the administration of IID-ThTPA-ICG NPs, indicating the clearance of the nanoparticles (Fig. S16). The body weight variations of the mice in different groups during the treatment (Fig. S17), H&E staining of the major organs of the mice in different groups after treatment (Fig. 5h), hematological index, and blood biochemical parameter analyses (Fig. 6a, b) all suggest there is no obvious systemic toxicity caused by IID-ThTPA NPs. Specifically, normal ALT, AST, BUN, and CREA levels indicate that liver and kidney functions of the mice are not affected after treatment, which fully demonstrates the biological safety of IID-ThTPA NPs.

## Conclusions

In summary, a novel isoindigo-based conjugated small molecule (IID-ThTPA) is synthesized via acceptor-oriented molecular design and prepared to colloidal IID-ThTPA NPs that exhibit competitive photothermal conversion efficiency (35.4%) and much enhanced singlet oxygen quantum yield (84.0%) as compared with the previous DPP-based nanoparticles. The ultrahigh singlet oxygen quantum yield of IID-ThTPA NPs originates from the narrow singlet–triplet energy gap of IID-ThTPA as revealed by DFT calculation. Moreover, with excellent colloidal stability and biocompatibility, efficient both in vitro and in vivo synergistic PTT/PDT can be achieved under a single NIR laser irradiation by using IID-ThTPA NPs. To the best of our knowledge, this is the first report of molecularly engineered conjugated small molecule nanoparticles with both high photothermal conversion efficiency and singlet oxygen quantum yield. This work not only demonstrates a highly efficient phototheranostic agent of IID-ThTPA NPs, but also provides a general molecular design strategy to manipulate the energy level of the excited states of conjugated small molecules for high-performance phototheranostics by taking advantage of the diversity of donors and acceptors.

## Electronic supplementary material

Below is the link to the electronic supplementary material.

Supplementary material 1 (DOCX 19130 kb)
