# Supplementary material for: Molecular Design of Conjugated Small Molecule Nanoparticles for Synergistically Enhanced PTT/PDT
Source: Nanomicro Lett. 2020 Jul 13;12:147. doi: 10.1007/s40820-020-00474-6 (PMC7770699; doi:10.1007/s40820-020-00474-6)
Supplement: Supplementary file 1 — Supplementary material 1 (DOCX 19130 kb) [file 40820_2020_474_MOESM1_ESM.docx]

Supporting Information for

**Molecular Design of Conjugated Small Molecule Nanoparticles for Synergistically Enhanced PTT/PDT**

Wei Shao^1, #^, Chuang Yang^1, 2, #^, Fangyuan Li^1,^ *, Jiahe Wu^1^, Nan Wang^1^, Qiang Ding^2^, Jianqing Gao^1^, Daishun Ling^1,^ *

^1^Institute of Pharmaceutics and Hangzhou Institute of Innovative Medicine, College of Pharmaceutical Sciences, Zhejiang University, Hangzhou, Zhejiang 310058, People’s Republic of China

^2^Jiangsu Breast Disease Center, The First Affiliated Hospital with Nanjing Medical University, Nanjing, Jiangsu 210029, People’s Republic of China

^#^Wei Shao and Chuang Yang contributed equally to this work.

*Corresponding authors. E-mail: [lfy@zju.edu.cn](mailto:lfy@zju.edu.cn) (F. L.); [lingds@zju.edu.cn](mailto:lingds@zju.edu.cn) (D. L.)

**S1 Experimental Section**

**S1.1 Measurements and Characterization**

^1^H and ^13^C nuclear magnetic resonance (NMR) spectra were recorded on a Bruker Avance III spectrometer operating at 400 and 125 MHz using CDCl_3_ as a solvent and tetramethylsilane (TMS) as an internal reference at room temperature. High-resolution matrix-assisted laser desorption/ionization time-of-flight (MALDI-TOF) mass spectroscopy was performed on a Bruker UltrafleXtreme mass spectrometer using 2,5-dihydroxybenzoic acid (DHB) as a matrix. Ultraviolet-visible-near-infrared (UV-Vis-NIR) absorption spectroscopy was performed on a UV-2600 spectrophotometer (Shimadzu, Japan). Fluorescence spectroscopy was performed on a Cary Eclipse fluorescence spectrophotometer (Agilent, USA). Digital photographs were taken by a smartphone. The morphology of IID-ThTPA NPs was characterized by transmission electron microscopy (TEM) (Hitachi HT7700, Japan). Dynamic light scattering (DLS) measurements were conducted on a Zetasizer Nano ZS90 equipment (Malvern Instruments, UK). Infrared thermographs were captured by a FLIR A350 thermal imaging camera. Photoacoustic imaging (PAI) was conducted on a Vevo LAZR system (Vevo 2100) (FUJIFILM VisualSonics, Canada). Confocal laser scanning microscopy (CLSM) images were acquired on a FLUOVIEW FV 1000 microscope (Olympus). The density functional theory (DFT) calculation was performed with the Gaussian 16 program package using the B3LYP functional with the 6-31G* basis set [S1]. The use of mice in this study was approved by the animal experimental ethics committee from Zhejiang University and all procedures were performed in accordance with the committee guidelines.

**S1.2 Materials**

Unless indicated, all commercially available reagents were used as received. Tetrahydrofuran (THF) and toluene were distilled under benzophenone and sodium prior to use. All the reactions were carried out under argon atmosphere.


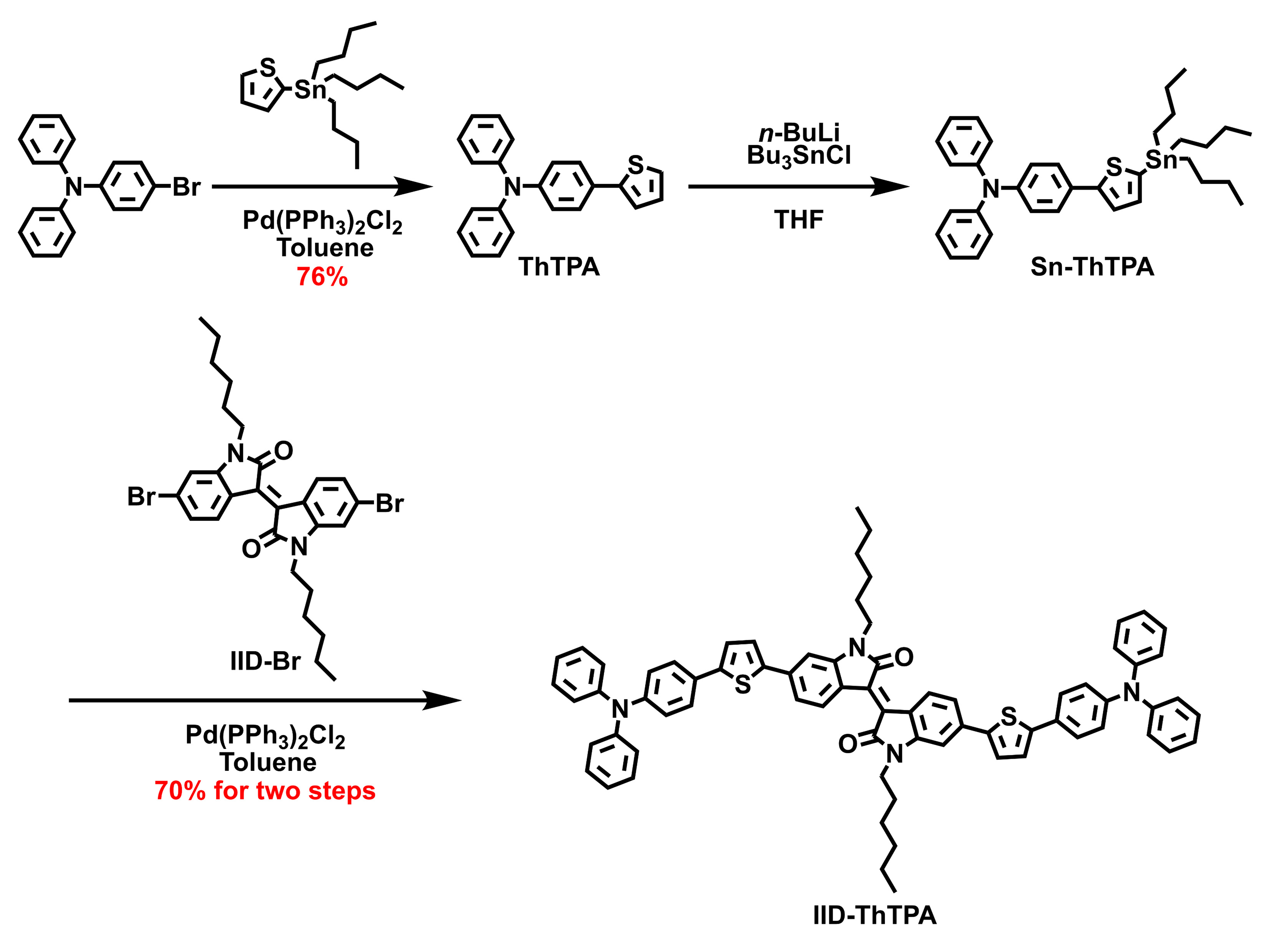


**Scheme S1** Synthesis of IID-ThTPA

**S1.3 Synthesis of ThTPA**

In a 100 mL three-necked flask, 4-bromotriphenylamine (695.4 mg, 2.14 mmol), 2-(tributylstannyl)thiophene (1.12 g, 3.00 mmol), and Pd(PPh_3_)_2_Cl_2_ (70.7 mg, 0.10 mmol) were dissolved in 30 mL dry toluene. After degassing by three freeze-pump-thaw cycles and filling back with argon, the reaction mixture was heated to reflux for 6 h. Afterwards, cold water was injected to quench the reaction, followed by extracting with chloroform/water for several times. All organic layers were collected, dried over anhydrous MgSO_4_, filtered, and concentrated by rotary evaporation in a reduced pressure. The residue was subjected to silica gel column using petroleum ether (PE)/dichloromethane (DCM) (4/1, V/V) as eluent to get compound ThTPA as a white solid. Yield: 498.5 mg (76%). ^1^H NMR (400 MHz, CDCl_3_, 25 ^o^C) *δ* (ppm): 7.48 (d, *J* = 6.8 Hz, 2H), 7.26 (t, *J* = 6.4 Hz, 4H), 7.22-7.21 (m, 2H), 7.12 (d, *J* = 6.4 Hz, 4H), 7.07 (d, *J* = 6.8 Hz, 2H), 7.05-7.01 (m, 3H). ^13^C NMR (125 MHz, CDCl_3_, 25 ^o^C) *δ* (ppm): 147.48, 147.19, 144.24, 129.27, 128.52, 127.95, 126.70, 124.41, 123.98, 123.75, 123.01, 122.20.

**S1.4 Synthesis of Sn-ThTPA**

ThTPA (325.1 mg, 1.00 mmol) was dissolved in 15 mL dry THF under the protection of argon. The solution was cooled down to -78 ^o^C using a dry ice-acetone bath, and *n*-BuLi (0.50 mL, 2.5 M in *n*-hexane, 1.25 mmol) was added dropwise. The solution was slowly warmed up to room temperature and stirred for 1 h. Afterwards, the mixture was cooled down to -78 ^o^C again, and tributyltin chloride (438.0 mg, 1.35 mmol) was added in one portion, and the reaction mixture was allowed to slowly warm up to room temperature and stirred overnight. Then, cold water was poured into the flask and the mixture was extracted with chloroform. Organic layers were combined, dried over anhydrous MgSO_4_, filtered and concentrated by rotary evaporation in a reduced pressure to afford a colorless oil. The obtained crude product was used for the next step without further purification.

**S1.5 Synthesis of IID-ThTPA**

IID-Br (134.4 mg, 0.23 mmol), Sn-ThTPA (308.3 mg, 0.50 mmol), and Pd(PPh_3_)_2_Cl_2_ (10.6 mg, 0.02 mmol) were added into a 50 mL flask. 20 mL dry toluene was injected into the flask to afford a homogeneous solution. After degassing by three freeze-pump-thaw cycles and filling back with argon, the solution was heated to reflux for 24 h. Cold water was added to quench the reaction and the mixture was extracted with chloroform. Organic layers were collected and combined, dried over anhydrous MgSO_4_, filtered and concentrated under reduced pressure to get the crude product, which was purified by silica gel column chromatography using PE/DCM (2/1, V/V) as eluent to afford IID-ThTPA as a dark brown solid. Yield: 174.1 mg (70%). ^1^H NMR (400 MHz, CDCl_3_, 25 ^o^C) *δ* (ppm): 9.15 (d, *J* = 6.8 Hz, 2H), 7.49 (d, *J* = 6.8 Hz, 4H), 7.36 (d, *J* = 3.2 Hz, 2H), 7.29-7.25 (m, 10H), 7.19 (d, *J* = 2.8 Hz, 2H), 7.13 (d, *J* = 6.0 Hz, 8H), 7.07-7.04 (m, 8H), 6.89 (s, 2H), 3.79 (t, *J* = 5.6 Hz, 4H), 1.75-1.69 (m, 4H), 1.44-1.33 (m, 12H), 0.89 (t, *J* = 5.6 Hz, 6H). ^13^C NMR (125 MHz, CDCl_3_, 25 ^o^C) *δ* (ppm): 168.25, 147.59, 147.33, 145.16, 144.97, 142.18, 137.61, 132.30, 131.55, 130.30, 129.34, 127.81, 126.47, 125.38, 124.64, 123.37, 123.26, 120.94, 118.75, 104.14, 40.03, 31.51, 27.54, 26.74, 22.57, 14.08. HRMS (MALDI-TOF): m/z calcd for [C_72_H_64_N_4_O_2_S_2_]^+^: 1080.4471; found: 1080.4425.

**S1.6 Preparation of IID-ThTPA NPs, IID-ThTPA-RITC NPs, and IID-ThTPA-ICG NPs**

Water-soluble IID-ThTPA NPs were prepared according to a reported method with some modifications [S2]. Typically, 2.0 mg of IID-ThTPA and 30 mg of Pluronic F127 were dissolved in 2.0 mL of THF and the solution was stirred for 30 min to afford a homogeneous solution. 10 mL of deionized water was added to the solution quickly and the resulting mixture was left to be stirred vigorously overnight to evaporate THF. The IID-ThTPA NPs were separated by centrifugation at 8000 rpm for 10 min to remove unencapsulated surfactant and dye and then redispersed in 10 mL of deionized water before characterization. The concentration of the IID-ThTPA NPs was determined by UV-Vis-NIR spectroscopy. A small amount of the IID-ThTPA NPs aqueous solution was 20 times-diluted with THF, then the UV-Vis-NIR absorption spectrum was quantified. Based on the calibration curve of IID-ThTPA in THF, the concentration of the THF solution is 7.3 μg mL^-1^. Thus, the concentration of the IID-ThTPA NPs is 146 μg mL^-1^ with a dye loading efficiency of 73%. The IID-ThTPA-RITC NPs and IID-ThTPA-ICG NPs were prepared by the same method as IID-ThTPA NPs with addition of 20% (mol%) of RITC (ICG) (molar ratio to IID-ThTPA) as a fluorescent label.

**S1.7 Measurement of the Photothermal Effect of IID-ThTPA NPs**

The aqueous solutions of IID-ThTPA NPs (1.0 mL) with different concentrations (0, 10, 20, 40, and 80 μg mL^-1^) were irradiated by a 671 nm continuous wave (CW) laser with a power density of 1.00 W cm^-2^ for 10 min. For the photothermal effect under different laser power densities, the aqueous solutions of IID-ThTPA NPs (1.0 mL, 80 μg mL^-1^) were irradiated by a 671 nm CW laser with different power densities (0.25, 0.50, 0.75, and 1.00 W cm^-2^) for 10 min. The temperatures were monitored by a thermal imaging camera.

**S1.8 Calculation of the Photothermal Conversion Efficiency (*η*) of IID-ThTPA NPs**

According to the work of Roper *et al*. [S3], the energy balance for the whole system is as follows:

$$\sum_{i} m_{i}C_{p,i}\frac{dT}{dt} =Q_{IID-ThTPA NPs}+Q_{Dis}-Q_{Surr} (S1)$$

where *m* and *C_p_* are the mass and heat capacity of solvent (water), *T* is the solution temperature, *Q_IID-ThTPA NPs_* is the energy input of IID-ThTPA NPs, *Q_Dis_* is the baseline energy input of the sample cell, and *Q_surr_* is the heat conducted away from the system surface by air.

The laser induced energy, *Q_IID-ThTPA NPs_*, represents the heat dissipated by electron-photon relaxation under the irradiation of a 671 nm laser:

$$Q_{IID-ThTPA NPs}= I\left( 1 -{10}^{-A_{\lambda}} \right)\eta(S2)$$

where *I* is the incident energy of the NIR laser (mW), *A_λ_* is the absorbance of the IID-ThTPA NPs at the NIR laser wavelength (*λ*) of 671 nm, and *η* is the photothermal conversion efficiency from the incident NIR laser energy to thermal energy. *Q_Dis_* represents the heat dissipated from the sample cell and it is measured to be 28.2 mW using pure water as control.

The energy transfer to air, *Q_Surr_*, is a temperature-dependent parameter, which is linear with the output of thermal energy:

$$Q_{Surr}= hS\left( T -T_{Surr} \right) (S3)$$

where *h* is the heat transfer coefficient, *S* is the surface area of the container, *T* is the temperature of the system surface, *T_Surr_* is the surrounding temperature (25 ^o^C).

Once the power density of NIR laser is defined, the heat input (*Q_IID-ThTPA NPs_* + *Q_Dis_*) will be finite. Since the heat output (*Q_Surr_*) increases along with the rise in temperature according to Eq. (S3), the temperature of system will reach a maximum when the heat output is equal to heat input:

$$Q_{IID-ThTPA NPs}+Q_{Dis}=Q_{Surr-Max}= hS\left( T_{Max}-T_{Surr} \right) (S4)$$

where *Q_Surr-Max_* represents conducting heat away from the system surface by air when the sample cell reaches the equilibrium temperature. *T_Max_* is the equilibrium temperature, representing no heat conduction away from the system surface by air. *η* can be obtained by substituting Eq. (S2) for *Q_IID-ThTPA NPs_* into Eq. (S4) and rearranging to get:

$$\eta= \frac{hS\left( T_{Max}-T_{Surr} \right) -Q_{Dis}}{I(1 -{10}^{-A_{\lambda}})} (S5)$$

where *A_λ_* is the absorbance of IID-ThTPA NPs at 671 nm. Thus, only *hS* remains unknown for calculating *η*.

In order to obtain *hS*, a dimensionless driving force temperature, *θ*, is introduced:

$$\theta= \frac{T -T_{Surr}}{T_{Max}-T_{Surr}} (S6)$$

and a time constant of sample system, *τ_s_*:

$$\tau_{s}= \frac{\sum_{i} m_{i}C_{p,i}}{hS} (S7)$$

which is substituted into Eq. (S1) and rearranged to yield:

$$\frac{d\theta}{dt} = \frac{1}{\tau_{s}}\left[ \frac{Q_{IID-ThTPA NPs}+ Q_{Dis}}{hS(T_{Max}- T_{Surr})} - \theta\right] (S8)$$

At the cooling period of IID-ThTPA NPs solution, the laser irradiation ceases, *Q_IID-ThTPA NPs_* + *Q_Dis_* = 0, reducing Eq. (S8) to:

$$dt = -\tau_{s}\frac{d\theta}{\theta} (S9)$$

and integrating gives the following expression:

$$t = -\tau_{s}\ln\theta(S10)$$

Therefore, the time constant of heat transfer from the system was determined to be 442.7 s for IID-ThTPA NPs. In addition, *m* is 1.0 g and *C* is 4.2 J g^-1^. Therefore *hS* can be determined. Substituting *hS* into Eq. (S5), *η* of the IID-ThTPA NPs can be calculated to be 35.4%.

**S1.9 Photostability Evaluation of IID-ThTPA NPs**

For photostability evaluation of IID-ThTPA NPs, aqueous solution of IID-ThTPA NPs (1.0 mL, 80 μg mL^-1^) was filled in a cuvette, then it was subjected to five laser (671 nm, 1.00 W cm^-2^) on/off cycles and the temperature was monitored by a thermal imaging camera. A clinically used photosensitizer indocyanine green (ICG) was used for comparison. IID-ThTPA NPs and ICG solutions were subjected to 671 nm laser irradiation at a power density of 1.00 W cm^-2^ for 20 min, and the UV-Vis-NIR absorption spectra were recorded every 5 min.

**S1.10 Electron Spin Resonance (ESR) Measurement**

ESR measurement was used to identify the type of ROS using 2,2,6,6-tetramethylpiperidine (TEMP) as the ^1^O_2_ indicator and 5-*tert*-butoxycarbonyl-5-methyl-1-pyrroline-*N*-oxide (BMPO) as the hydroxyl radical and superoxide anion radical indicator. Samples were prepared by mixing 200 μL of IID-ThTPA NPs (200 μg mL^-1^) in water and 200 μL of TEMP or BMPO (100 mM) in water. ESR signals were recorded by adding samples through a capillary tube under a 671 nm laser irradiation at 1.00 W cm^-2^ (0, 1, 2, and 3 min).

**S1.11 Calculation of the Singlet Oxygen Quantum Yield (Φ_Δ_) of IID-ThTPA NPs**

Calculation of the Φ_Δ_ is based on a reported method [S4]. 1,3-diphenylisobenzofuran (DPBF) was used as the ^1^O_2_-trapping agent, and methylene blue (MB) was used as the standard reference photosensitizer. Typically, 60 μL of DPBF solution in DMF (1.0 mg mL^-1^) was added to 1.5 mL of IID-ThTPA NPs aqueous solution, and a 671 nm laser was employed as the irradiation source. The absorbance of IID-ThTPA NPs and MB at 671 nm was set to be ~0.2. The absorbance of DPBF at 410 nm was recorded at various irradiation times to obtain the decay rate of DPBF under oxidation by the generated singlet oxygen. The Φ_Δ_ of IID-ThTPA NPs (*Φ_Δ,IID-ThTPA NPs_*) was calculated using the following formula:

$$\Phi_{\Delta, IID-ThTPA NPs}=\Phi_{\Delta,MB}\frac{t_{MB}}{t_{IID-ThTPA NPs}} (S11)$$

where *t_MB_* is the time for the decrease in the absorbance of DPBF in the presence of MB free in water adjusted to a first-order exponential decay, *t_IID-ThTPA NPs_* is the time for the decrease in the absorbance of DPBF in the presence of IID-ThTPA NPs in water adjusted to a first-order exponential decay, and *Φ_Δ,MB_* is the Φ_Δ_ of MB free in water given as 52.0% [S5].

**S1.12 Singlet Oxygen Sensor Green (SOSG) Assay for ^1^O_2_**

3 mL aqueous solution containing IID-ThTPA NPs (15 μg mL^-1^) and SOSG (20 μM) was added into a cuvette and irradiated with a 671 nm laser at a power density of 1.00 W cm^-2^ for different time intervals. The fluorescence spectrum of the solution was acquired in the range of 500-650 nm with the excitation wavelength of 488 nm. 3 mL aqueous solution only containing SOSG (20 μM) was treated under the same experimental condition for comparison as a control.

**S1.13 *In Vitro* Cytotoxicity Assay**

4T1 cells (1 × 10^4^ cells per well) were seeded in 96-well plates. After growing for 12 h, the cells were incubated with increasing concentrations of IID-ThTPA NPs (0, 5, 10, 20, 40, and 80 μg mL^-1^) for 24 h. Then MTT solution (31.5 μL per well, 5 mg mL^-1^ in PBS) was added. After incubation for another 4 h, the culture medium was replaced with DMSO (200 μL per well). A microplate reader (Bio-Rad, Hercules, California, USA) was used to detect the absorbance at 490 nm.

**S1.14 *In Vitro* Phototherapy**

For synergistic PTT/PDT: 4T1 cells (1 × 10^4^ cells per well) were firstly seeded in 96-well plates for 12 h before treatment. The culture medium was removed, and then IID-ThTPA NPs with different concentrations (0, 5, 10, 20, 40, and 80 μg mL^-1^) were added into the wells (100 μL per well). After 6 h of incubation, these wells were irradiated for 5 min under a 671 nm laser with a power density of 1.00 W cm^-2^. Finally, a standard MTT assay was used to evaluate the viability of the cells (n = 6).

For PTT only: 4T1 cells were co-incubated with a ROS scavenger Vitamin C (Vc) (0.5 mM) to exclude the PDT effect.

For PDT only: 4T1 cells were kept at 4 ^o^C during laser irradiation to avoid temperature elevation (the cells in cell culture plate were kept on ice and cooled to 4 ^o^C).

**S1.15 Cellular Uptake**

4T1 cells were seeded in CLSM dishes and allowed to adhere overnight. Then the cells were treated with the medium containing IID-ThTPA-RITC NPs (80 μg mL^-1^). After further incubation for certain time periods (1, 2, 4, and 6 h), the cells in each dish were rinsed with PBS for three times and then stained with DAPI (to label nuclei) and finally observed under a CLSM instrument.

**S1.16 Intracellular ROS Detection**

2’,7’-dichlorofluorescein diacetate (DCFH-DA, 10 μM) was used to stain 4T1 cells under different treatments: control (without treatment), IID-ThTPA NPs (IID-ThTPA NPs, 80 μg mL^-1^), laser (671 nm, 1.00 W cm^-2^, 5 min), and IID-ThTPA NPs + laser (IID-ThTPA NPs, 80 μg mL^-1^ + 671 nm, 1.00 W cm^-2^, 5 min) and the cells were observed under a CLSM instrument. The green fluorescence intensity of DCF indicates the intracellular ROS level.

**S1.17 *In Vitro* and *In Vivo* PAI**

For *in vitro* PAI, PA spectra of IID-ThTPA NPs with different concentrations (200, 400, 600, 800, and 1000 μg mL^-1^) were acquired between 680-900 nm. The PA intensities of these IID-ThTPA NPs with different concentrations at 680 nm were used to evaluate the linearity of the PA signal intensity as a function of IID-ThTPA NPs concentration.

For *in vivo* PAI, the 4T1 tumor-bearing mice were intravenously injected with IID-ThTPA NPs (200 μL, 800 μg mL^-1^). After injection, PA image at tumor site was acquired at different time points (0, 2, 4, 6, 12, and 24 h) under 680 nm pulsed laser excitation.

**S1.18 *In Vivo* Pharmacokinetics Study**

The mice (n = 3) were intravenously injected with IID-ThTPA-RITC NPs (200 μL, 800 μg mL^-1^ based on IID-ThTPA NPs). The blood was taken from mouse eyelids at different time points, then the blood was centrifugated to obtain the serum and the concentration of IID-ThTPA-RITC NPs in serum (12 times-diluted) was calculated according to the calibration curve. The calibration curve was obtained by plotting the FL intensity of IID-ThTPA-RITC NPs *versus* its concentration (based on IID-ThTPA NPs) in serum. The FL intensity was determined by a VIEWORKS VISQUE InVivo Elite equipment (Republic of Korea).

**S1.19 *In Vivo* Phototherapy**

The male Balb/c nude mice (~17 g) were obtained and raised at the Laboratory Animal Center of Zhejiang University. To develop the tumor model, 4T1 cells (1 × 10^6^ cells per mouse) suspended in 100 μL PBS were injected into the mice. *In vivo* phototherapy by intravenous administration was performed when the tumor volume reached ~100 mm^3^. All mice were anesthetized before NIR laser irradiation. The tumor site was irradiated with a 671 nm laser at 6 h post-injection of IID-ThTPA NPs (1.00 W cm^-2^, 5 min for the groups of laser, IID-ThTPA NPs + Vc + laser (PTT), and IID-ThTPA NPs + laser (PTT + PDT)) and the temperature of the tumor site was monitored by a thermal imaging camera (the ROS scavenger Vc was given by intratumoral injection at the dose of 25.0 μmol kg^-1^ before laser irradiation). The measurements of mice body weights and tumor volumes were conducted by a balance and digital caliper every two days during the entire treatment period. The tumor volume was calculated according to the following formula: tumor volume = (tumor length) × (tumor width)^2^ / 2. The weights of tumors of the mice in different groups were also measured at the end of treatments. Moreover, the hematoxylin and eosin (H&E), Ki-67, and TUNEL staining of the tumors was carried out to evaluate the tissue destruction and cell apoptosis after different treatments.

**S1.20 Biodistribution of IID-ThTPA-ICG NPs**

The mice (n = 3) were intravenously injected with IID-ThTPA-ICG NPs (200 μL, 800 μg mL^-1^ based on IID-ThTPA NPs). *Ex vivo* tissue and tumor fluorescence imaging was conducted at 6 h, 1 d, and 3 d post-injection.

**S1.21 *In Vivo* Toxicity Assay**

After 14 days of treatment, the mice in different groups were sacrificed. The major organs (heart, liver, spleen, lung, and kidneys) and tumor were excised and fixed in 4% formalin. Paraffin embedded sections were prepared and subjected to H&E staining. The slices were then examined by a digital microscope (Eclipse, Ti-S, Nikon, Japan). For the hematological index and biochemical blood analysis, healthy mice were divided into three groups randomly (control group, 7 days post-injection of IID-ThTPA NPs group, and 14 days post-injection of IID-ThTPA NPs group (200 μL, 800 μg mL^-1^), n = 3). The blood was collected at 7 and 14 days post-injection for analysis. For hematological index analysis, eight blood parameters were tested, including white blood cells (WBC), red blood cells (RBC), hemoglobin (HGB), hematocrit (HCT), mean corpuscular hemoglobin (MCH), mean corpuscular hemoglobin concentration (MCHC), mean corpuscular volume (MCV), and platelets (PLT). For biochemical blood analysis, five parameters were tested, including alanine transaminase (ALT), aspartate transaminase (AST), blood urea nitrogen (BUN), creatinine (CREA), and total bilirubin (TBIL).

**S2 Supporting Figures and Tables**


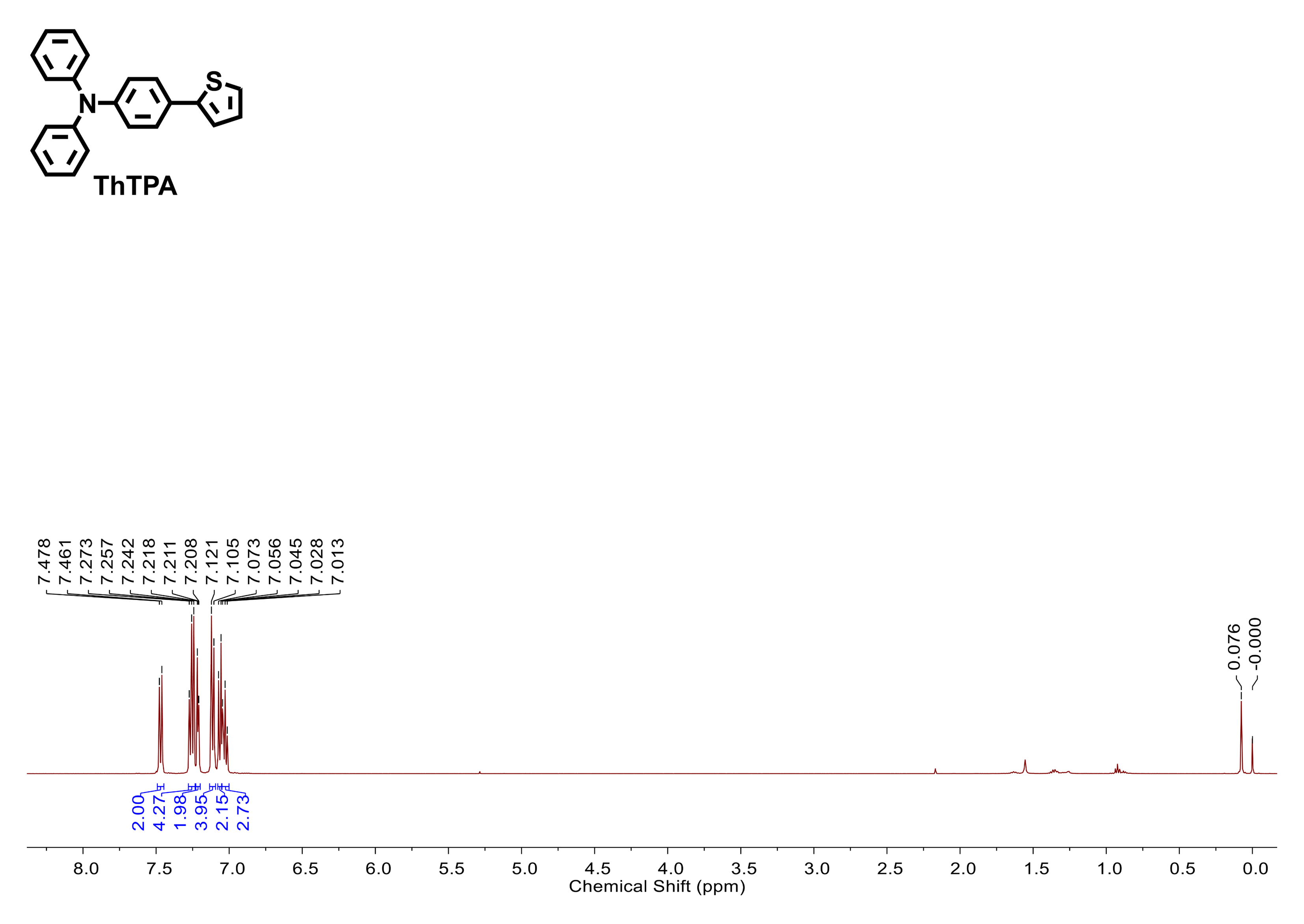


**Fig. S1** ^1^H NMR spectrum of ThTPA (CDCl_3_, 25 ^o^C)


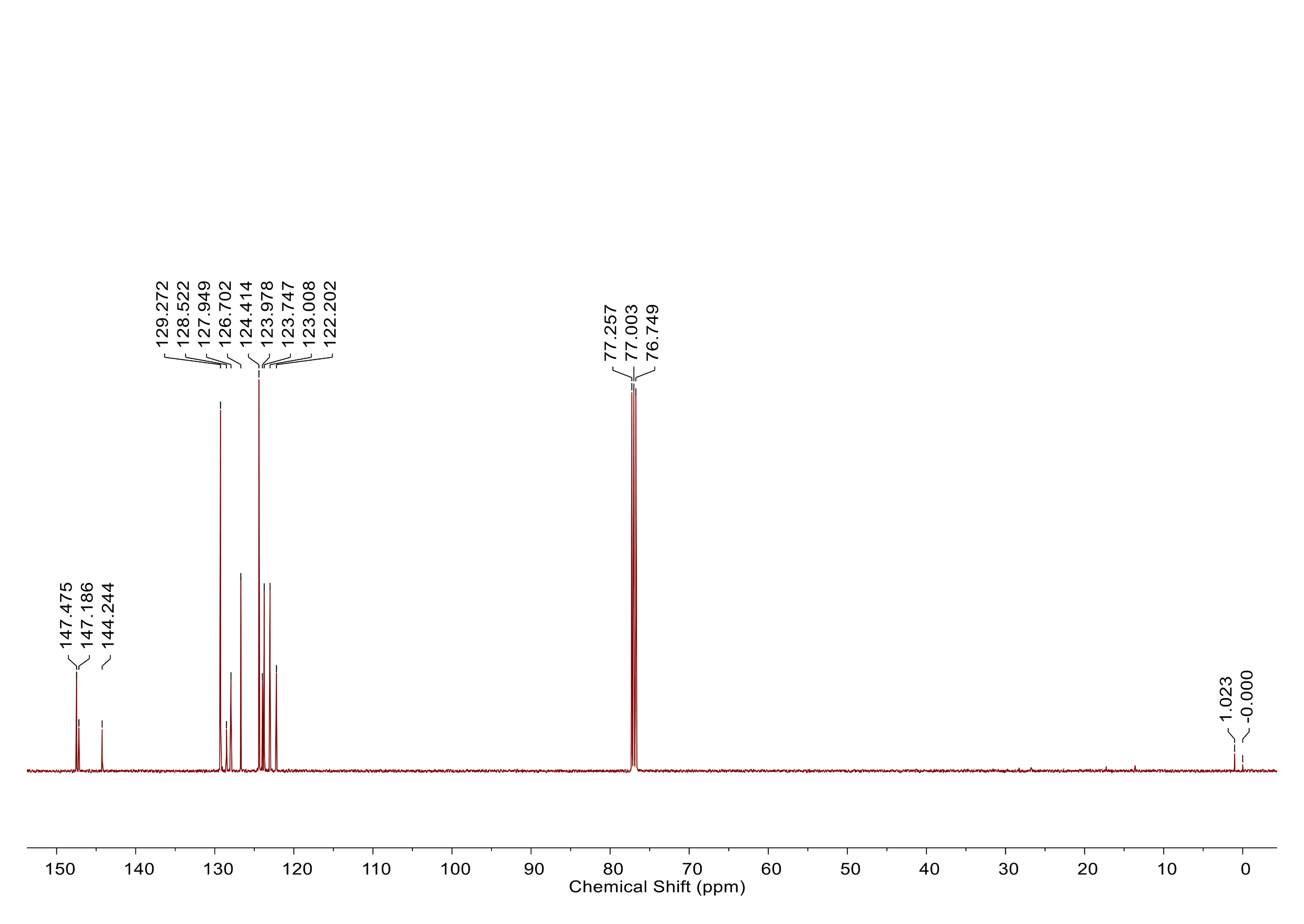


**Fig. S2** ^13^C NMR spectrum of ThTPA (CDCl_3_, 25 ^o^C)


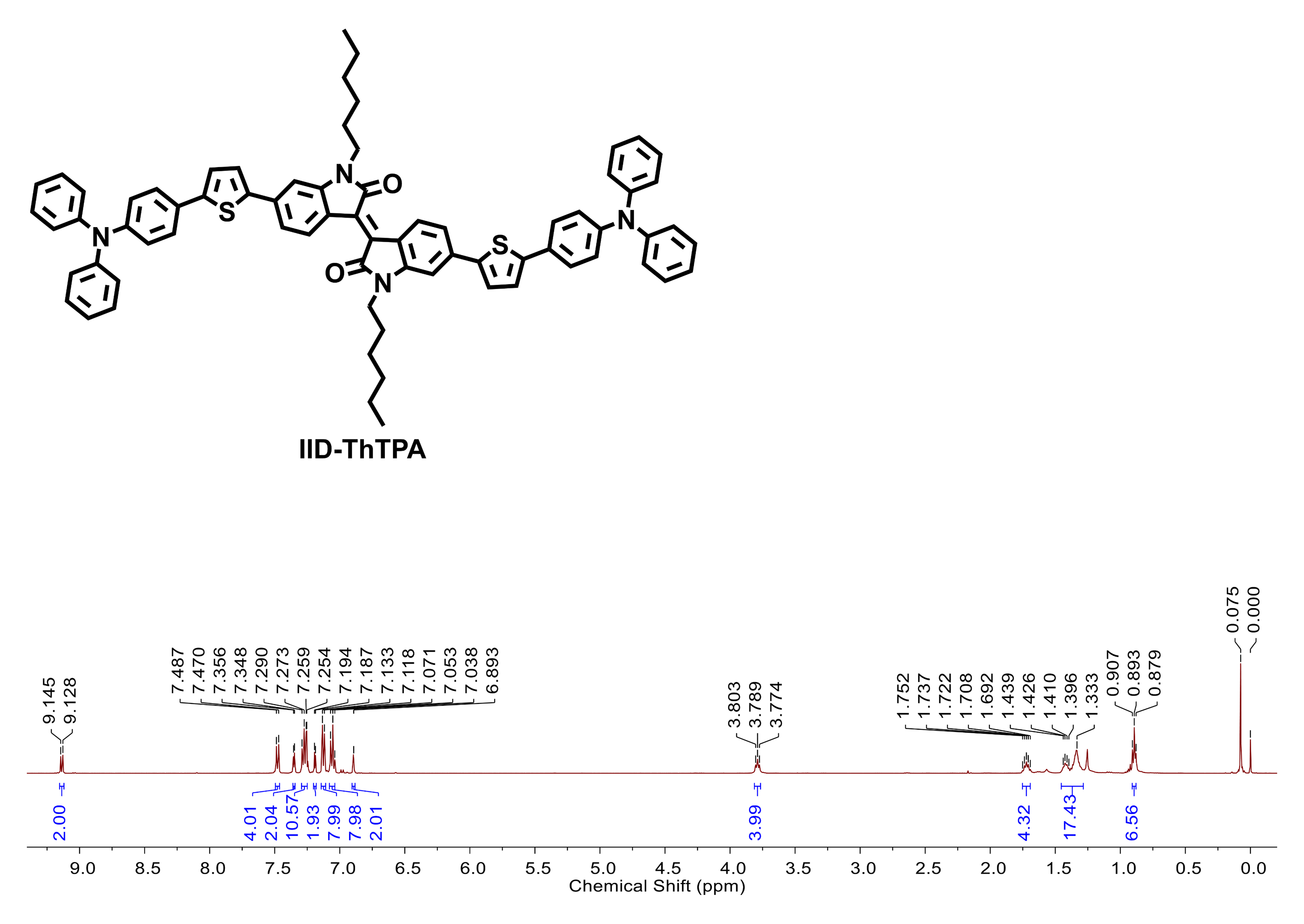


**Fig. S3** ^1^H NMR spectrum of IID-ThTPA (CDCl_3_, 25 ^o^C)


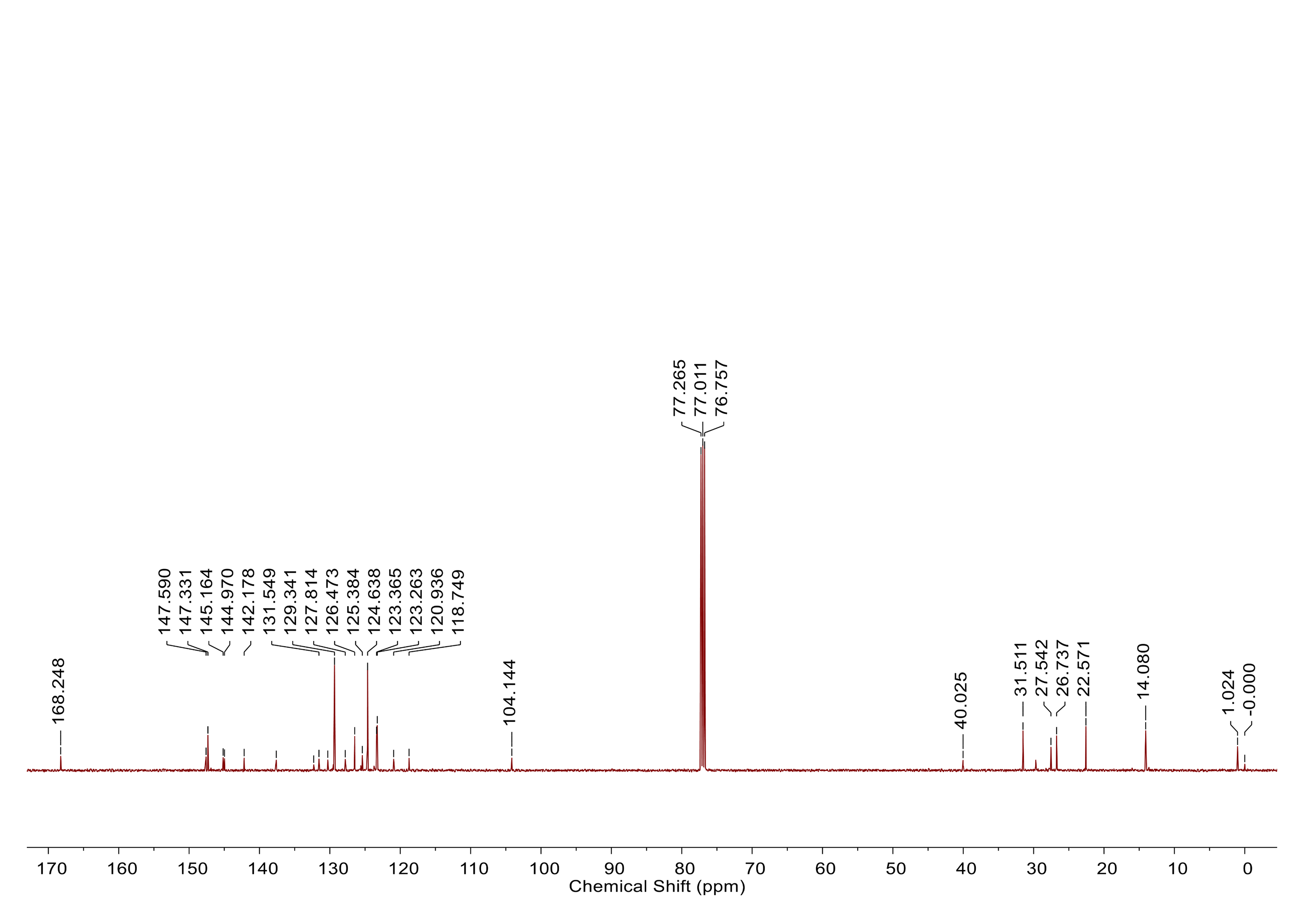


**Fig. S4** ^13^C NMR spectrum of IID-ThTPA (CDCl_3_, 25 ^o^C)


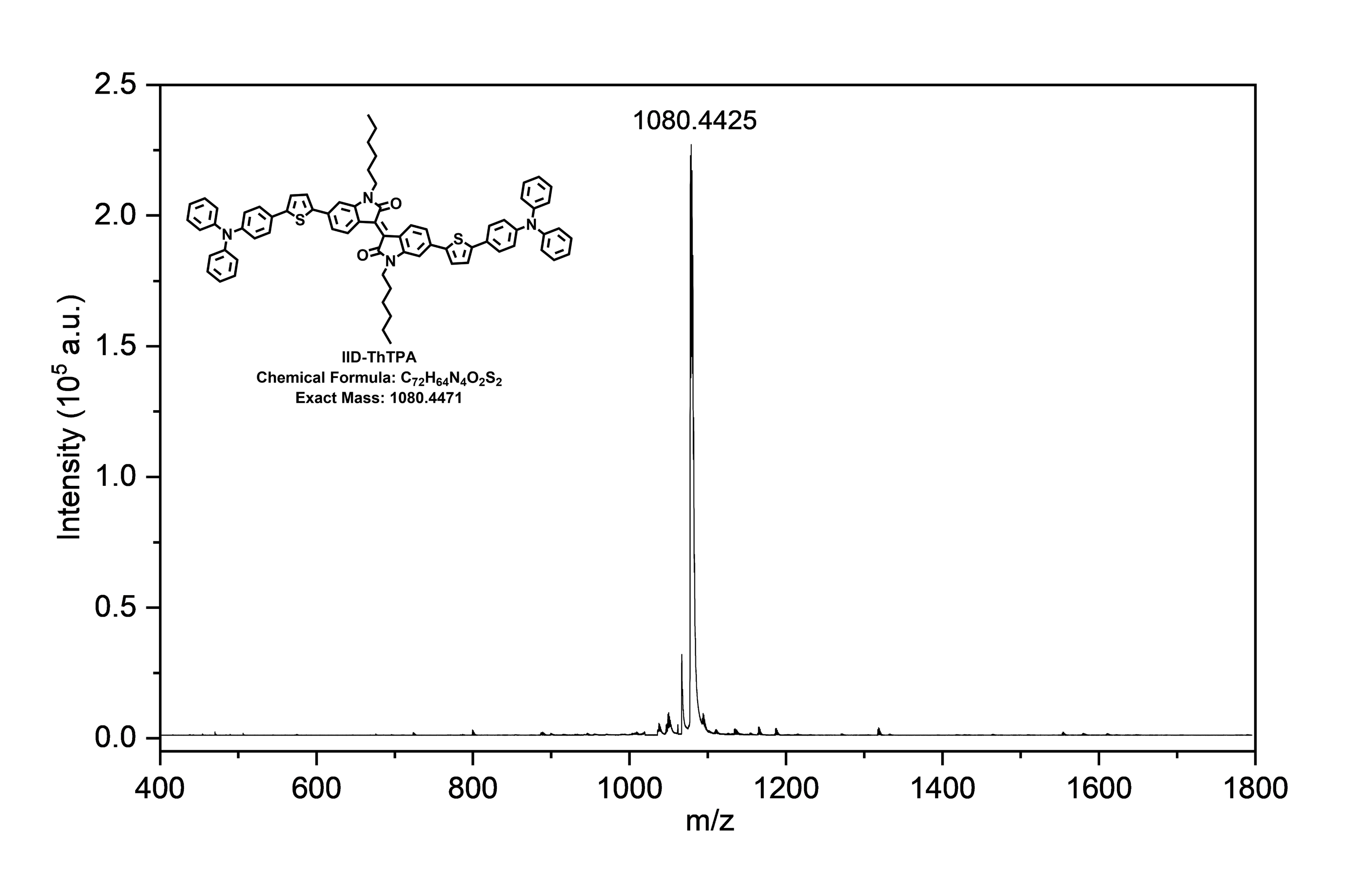


**Fig. S5** High-resolution MALDI-TOF mass spectrum of IID-ThTPA


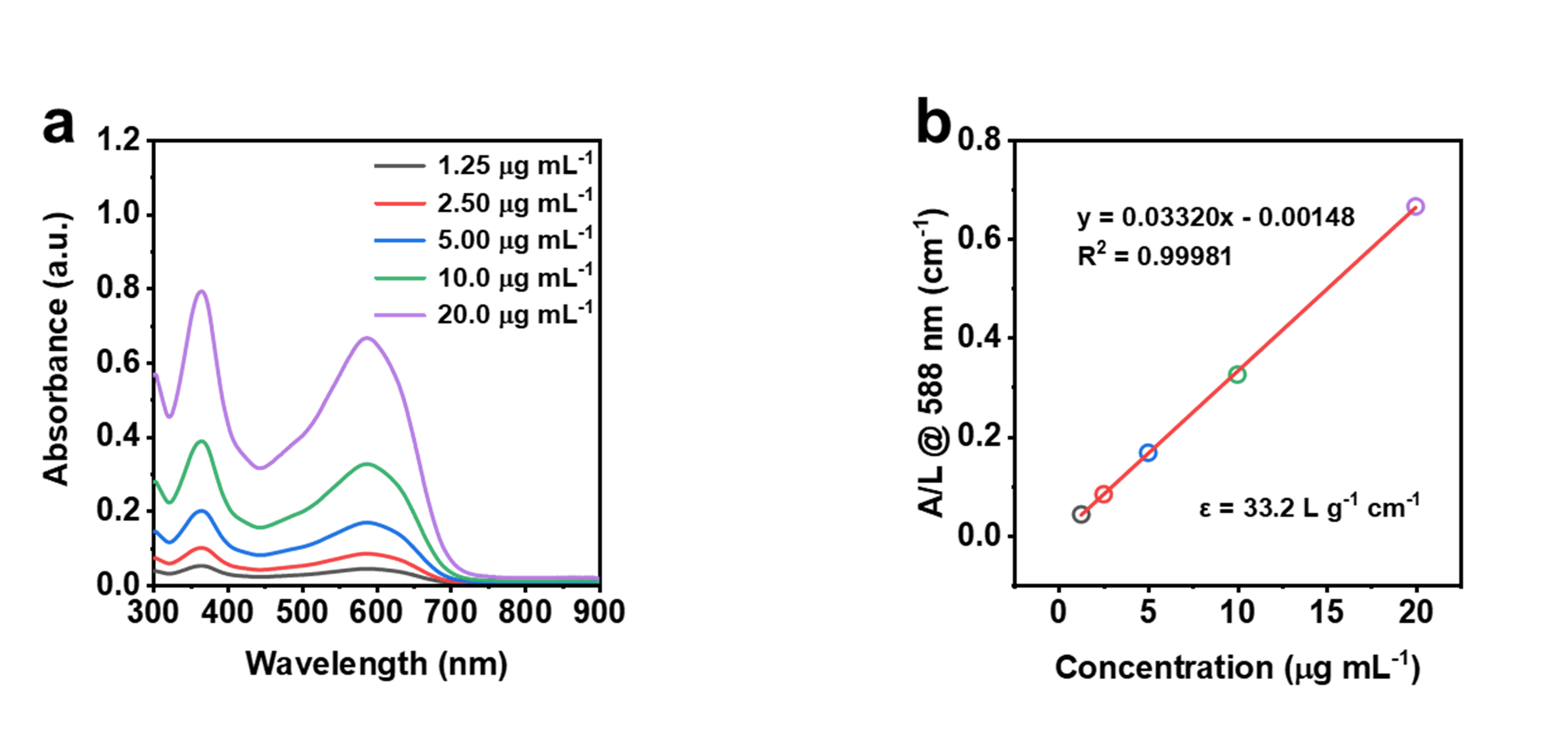


**Fig. S6** (**a**) UV-Vis-NIR absorption spectra of IID-ThTPA in THF with different concentrations. (**b**) The calibration curve of IID-ThTPA in THF


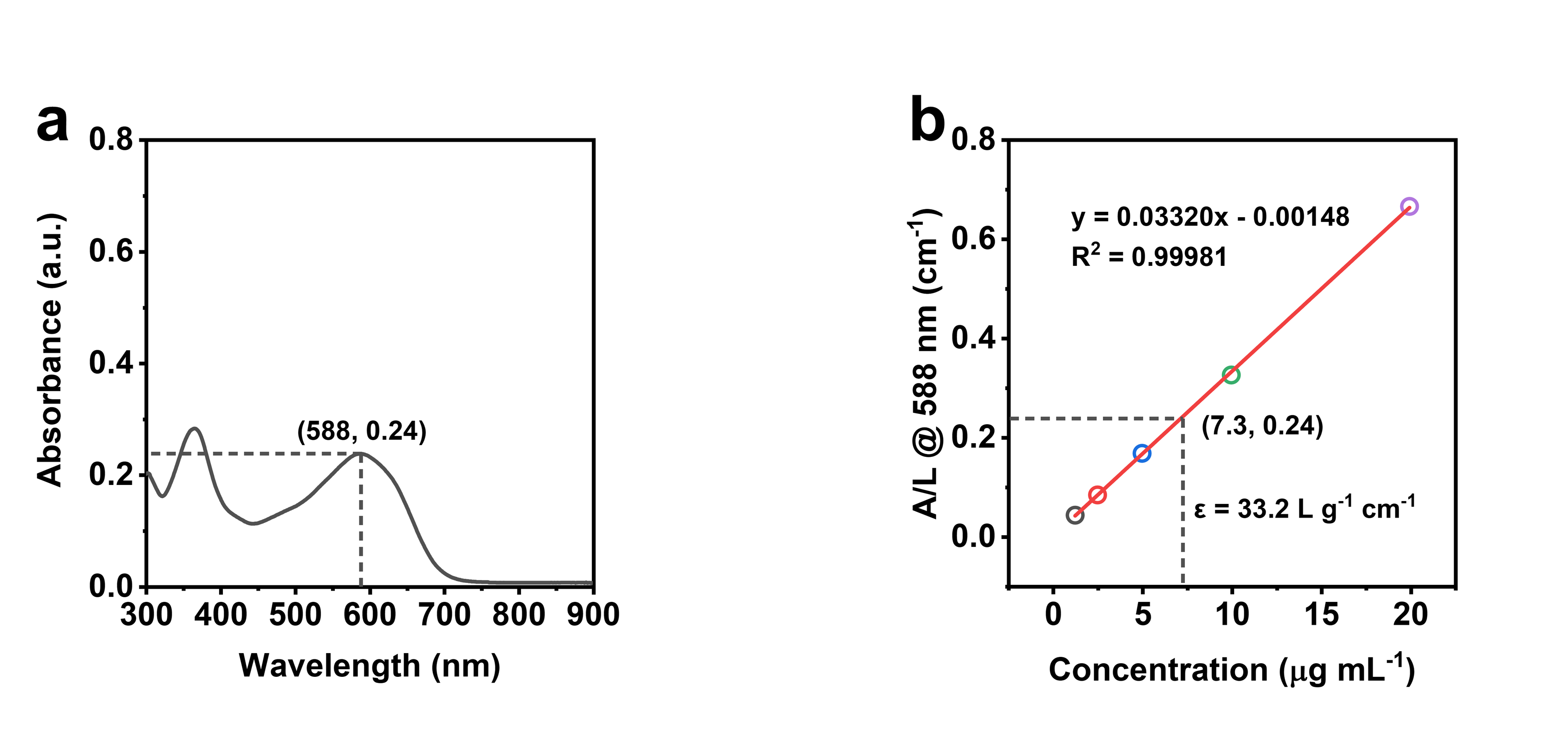


**Fig. S7** Determination of the concentration of IID-ThTPA NPs. (**a**) UV-Vis-NIR absorption spectrum of the 20 times-diluted THF solution of IID-ThTPA NPs. (**b**) Concentration determination of the THF solution based on the calibration curve


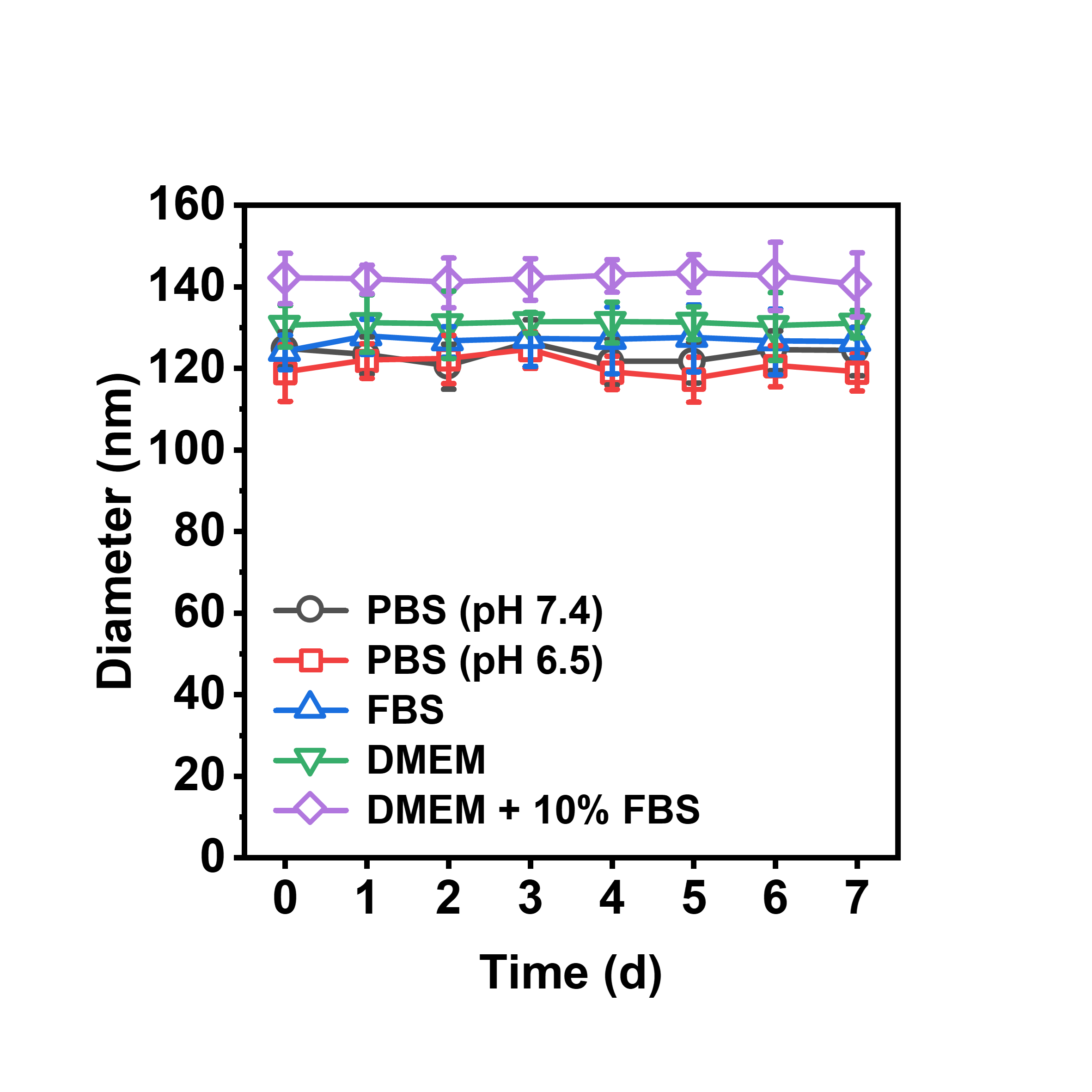


**Fig. S8** Size changes of IID-ThTPA NPs in various mediums during one week’s storage at room temperature (the results are presented as mean ± SD, n = 3)


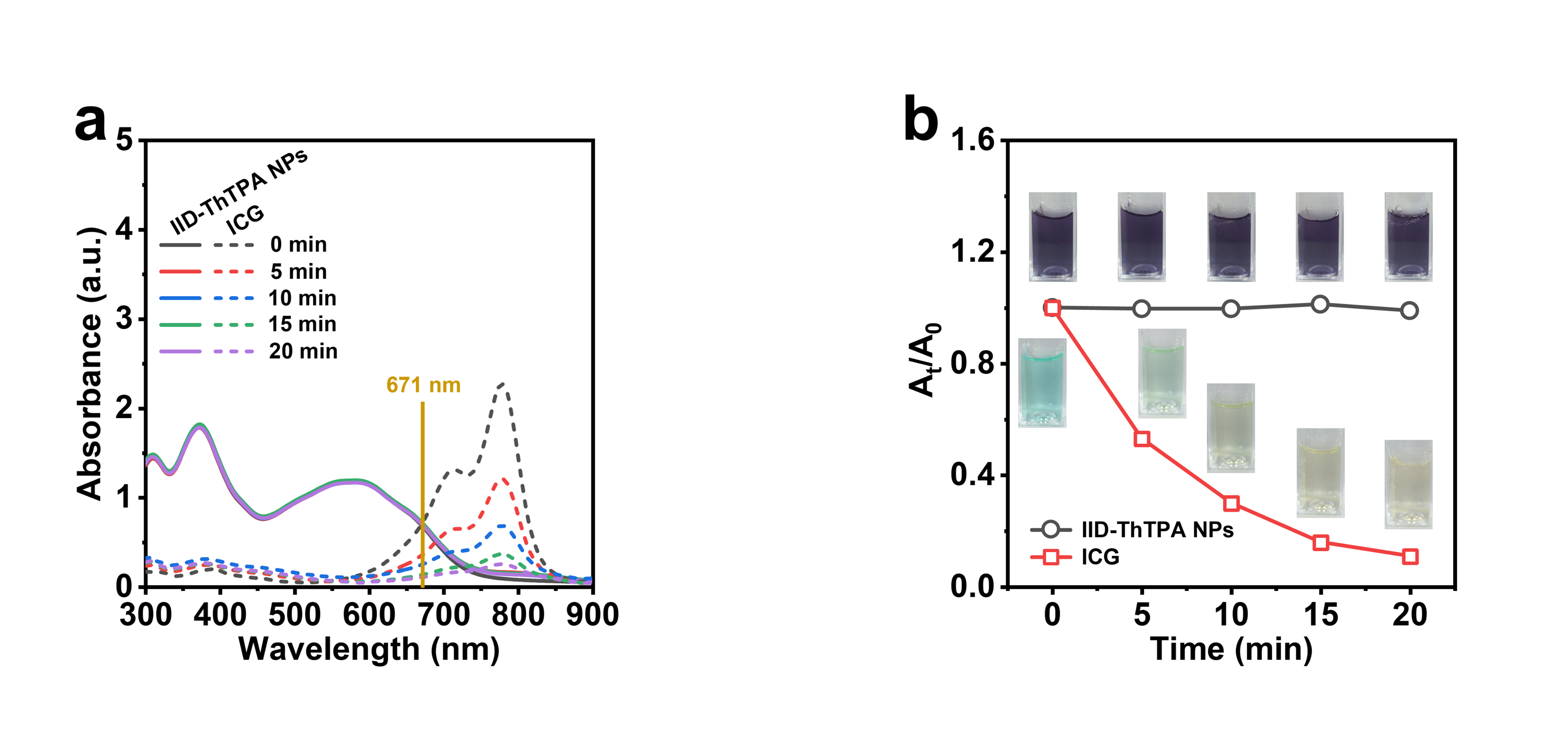


**Fig. S9** (**a**) UV-Vis-NIR absorption spectra of IID-ThTPA NPs and ICG solutions after 671 nm laser irradiation for different times. (**b**) A_t_/A_0_ of IID-ThTPA NPs and ICG solutions *versus* 671 nm laser irradiation time (insets are the digital photographs of IID-ThTPA NPs and ICG solutions after 671 nm laser irradiation for different times)


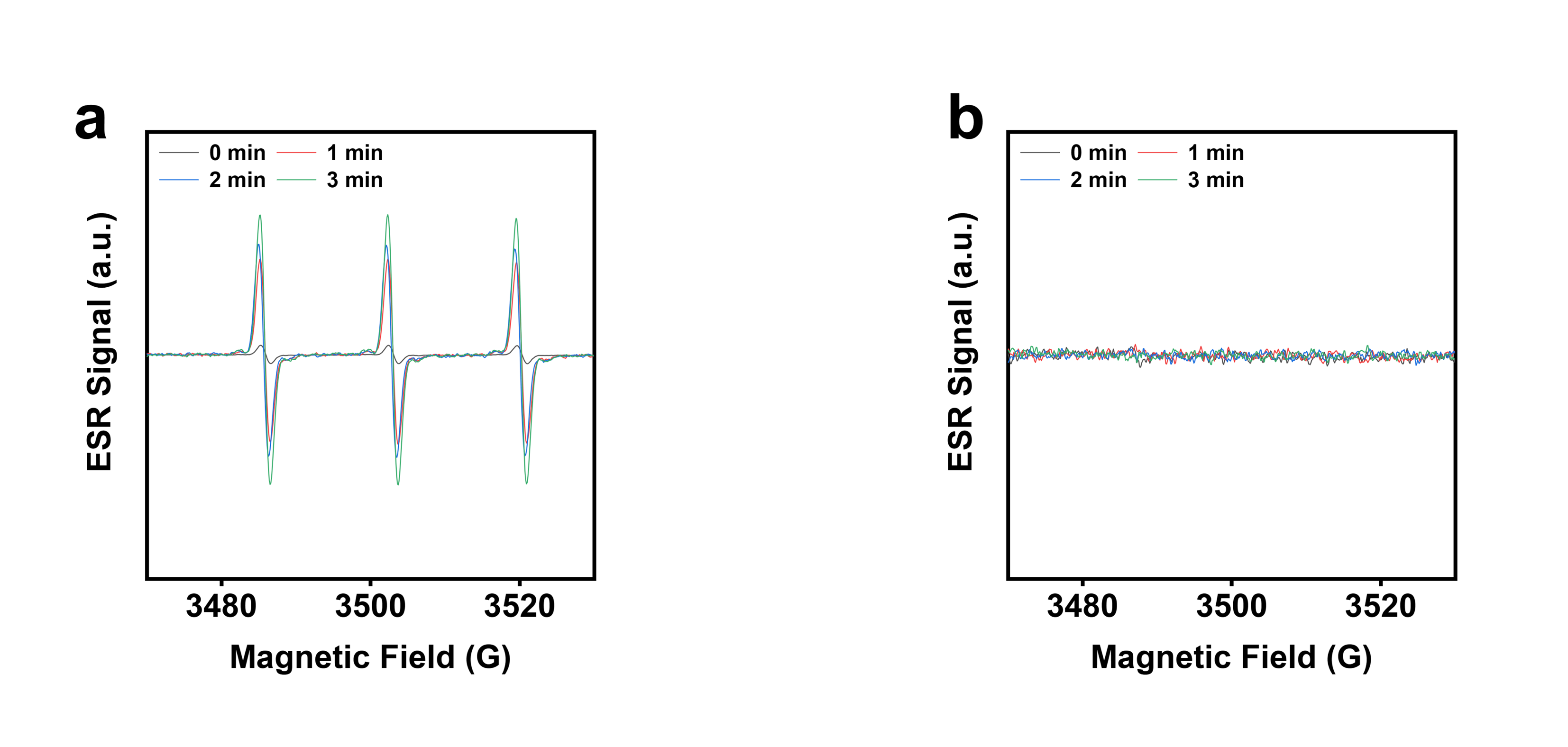


**Fig. S10** (**a**) ESR spectra of TEMP in the presence of IID-ThTPA NPs under laser irradiation for different times. (**b**) ESR spectra of BMPO in the presence of IID-ThTPA NPs under laser irradiation for different times


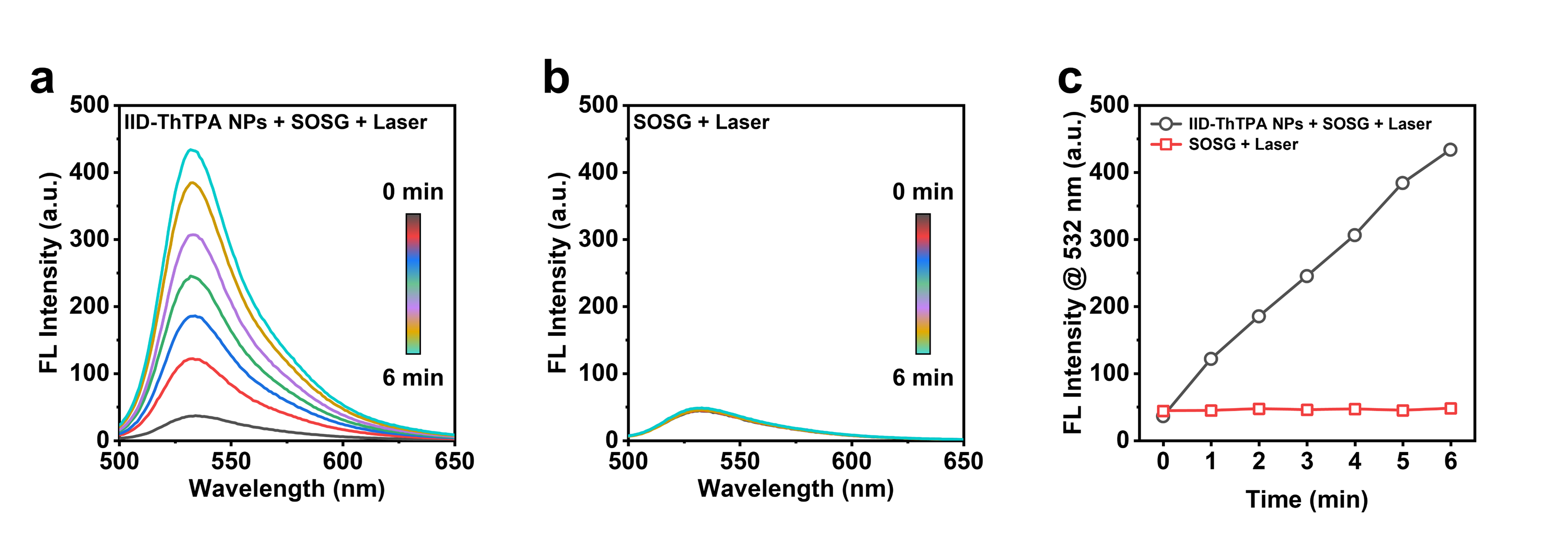


**Fig. S11** (**a**) Fluorescence spectra of SOSG in the presence of IID-ThTPA NPs under laser irradiation for different times. (**b**) Fluorescence spectra of SOSG in the absence of IID-ThTPA NPs under laser irradiation for different times. (**c**) Plot of SOSG fluorescence intensity at 532 nm *versus* time in (**a**) and (**b**)


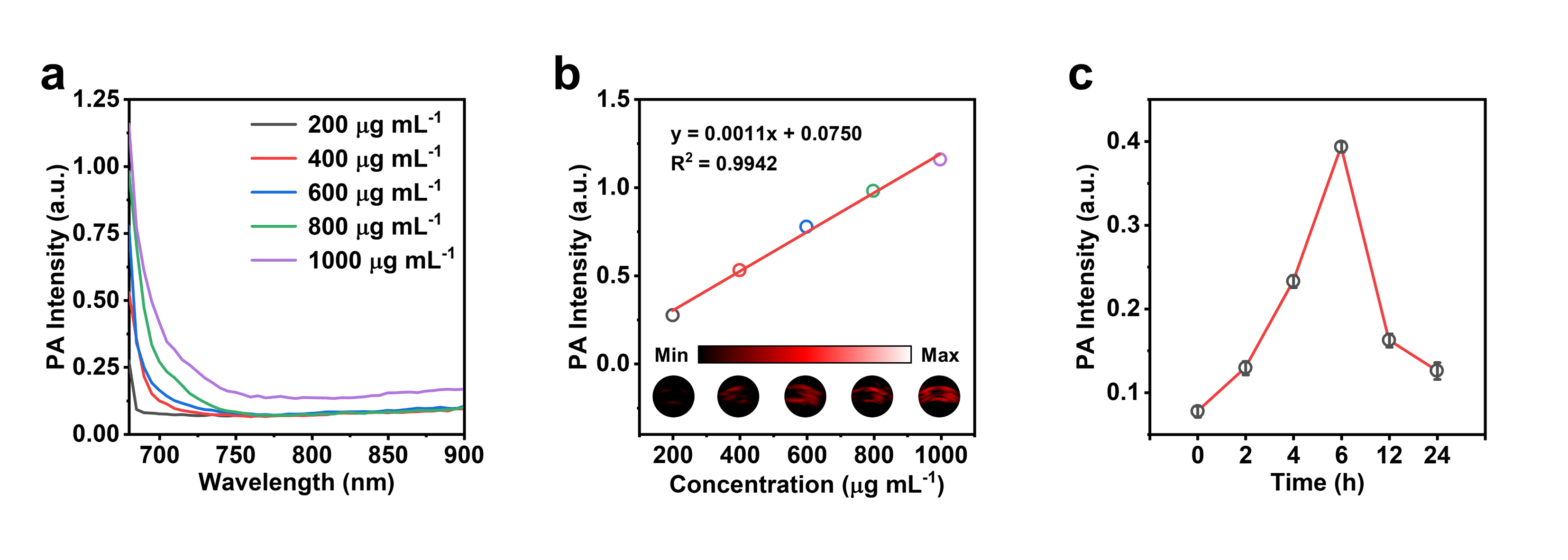


**Fig. S12** (**a**) PA spectra of IID-ThTPA NPs with different concentrations (200, 400, 600, 800, and 1000 μg mL^-1^). (**b**) Linear plot of PA intensities of IID-ThTPA NPs at 680 nm *versus* their concentrations (insets are the PA images of IID-ThTPA NPs at 680 nm with different concentrations). (**c**) PA intensities of the tumor sites at 680 nm at different time points post-injection (0, 2, 4, 6, 12, and 24 h) (the results are presented as mean ± SD, n = 3)


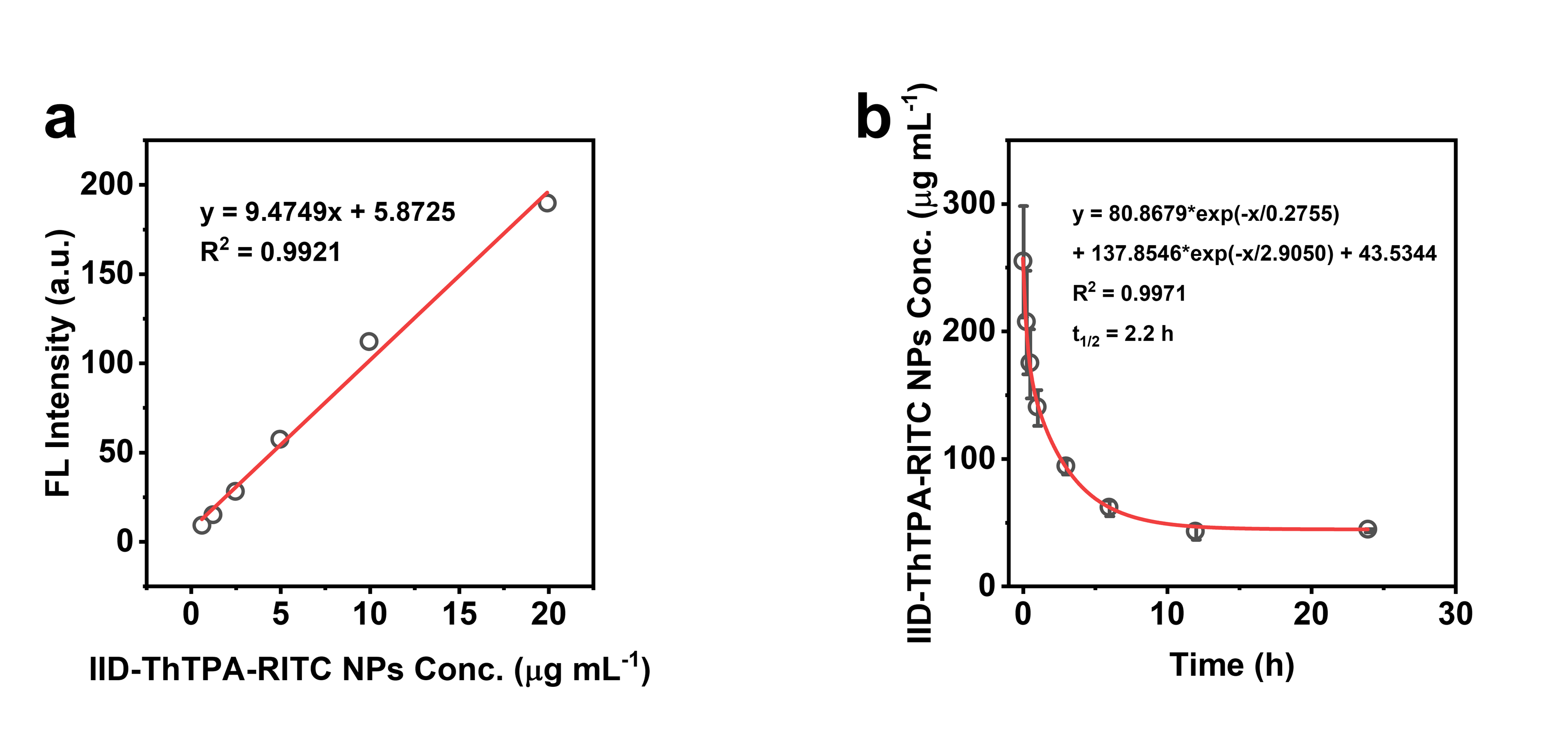


**Fig. S13** (**a**) The fitted calibration curve of FL intensity *versus* concentration of IID-ThTPA-RITC NPs (based on IID-ThTPA NPs) in serum. (**b**) Pharmacokinetics profile of IID-ThTPA-RITC NPs after intravenous injection into mice (the results are presented as mean ± SD, n = 3)


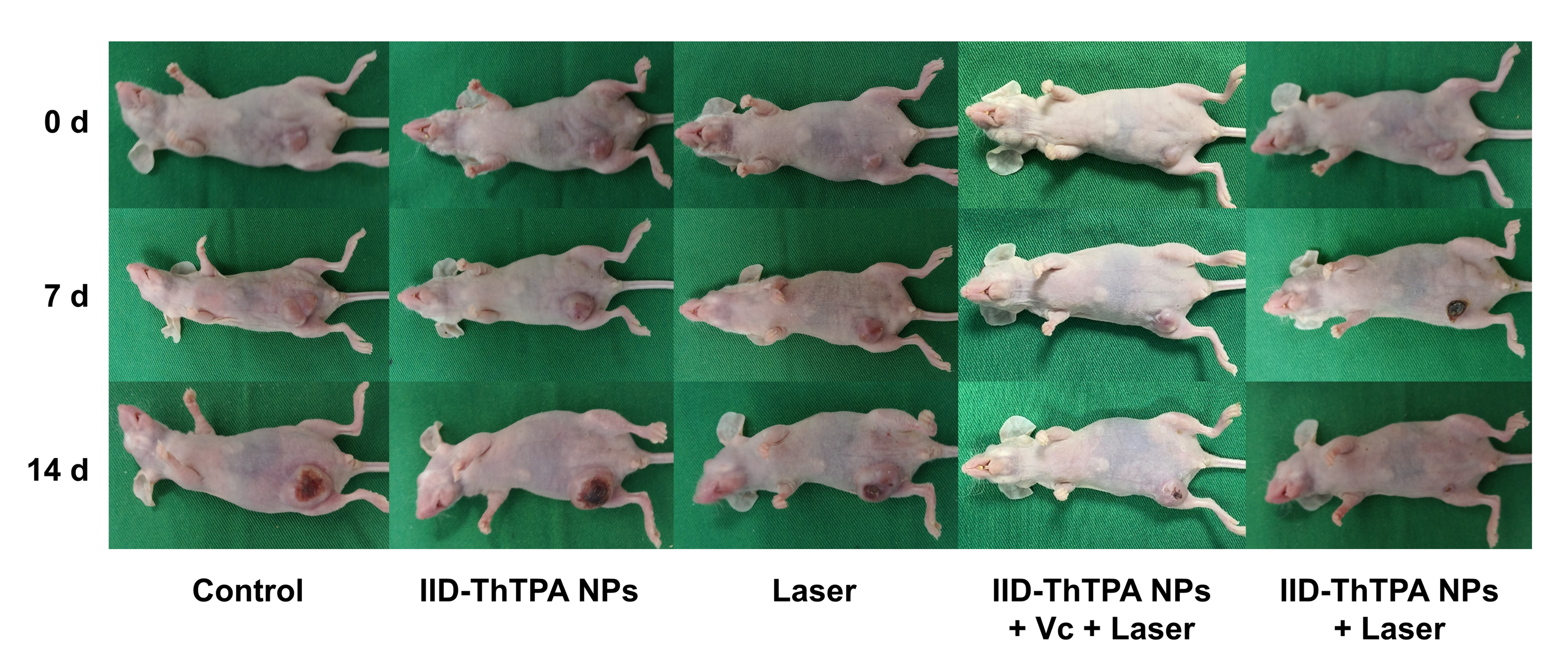


**Fig. S14** Digital photographs of the mice in control, IID-ThTPA NPs, laser, IID-ThTPA NPs + Vc + laser (PTT), and IID-ThTPA NPs + laser (PTT + PDT) groups at 0, 7, and 14 d


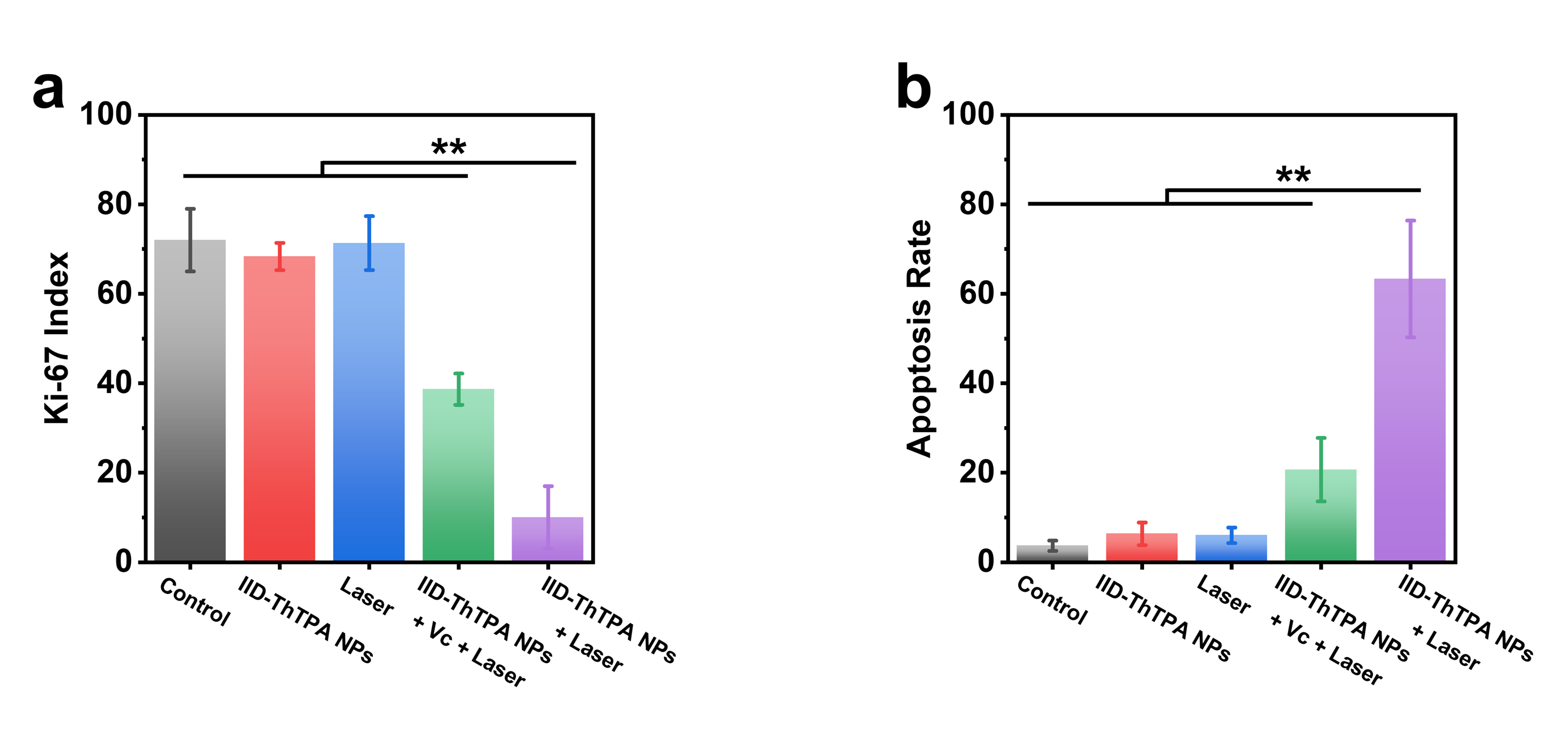


**Fig. S15** (**a**) The Ki-67 index in different groups. (**b**) The apoptosis rate in different groups (**P < 0.01)


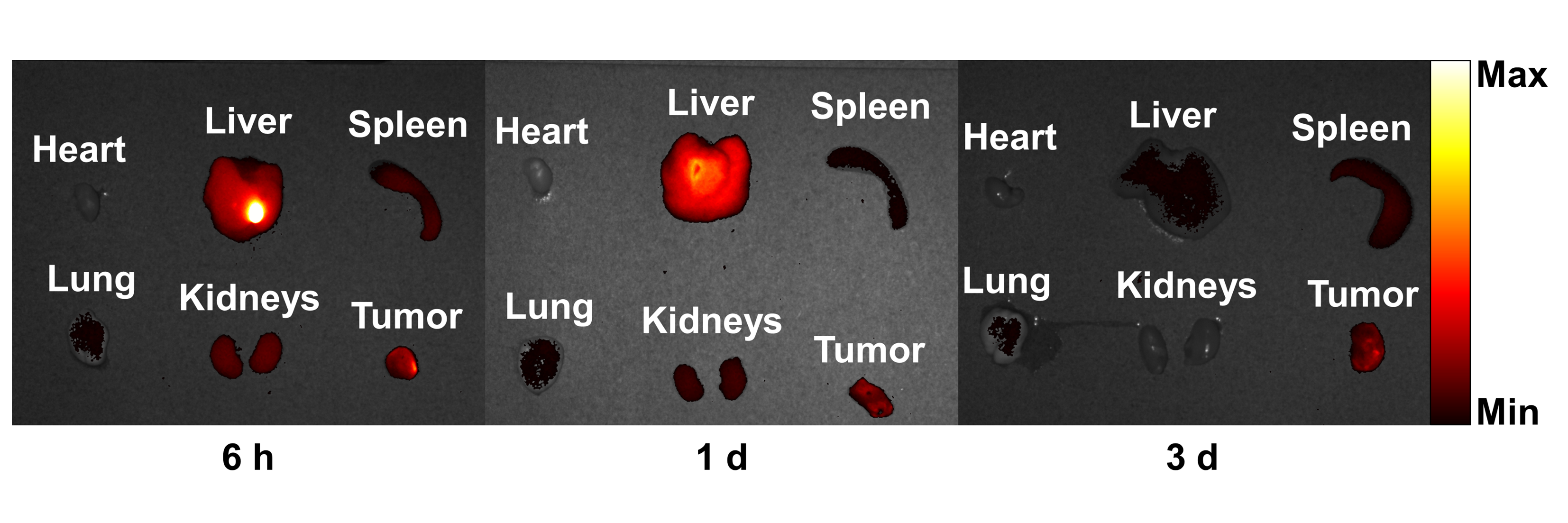


**Fig. S16** *Ex vivo* tissue and tumor fluorescence imaging at different times post-injection of IID-ThTPA-ICG NPs


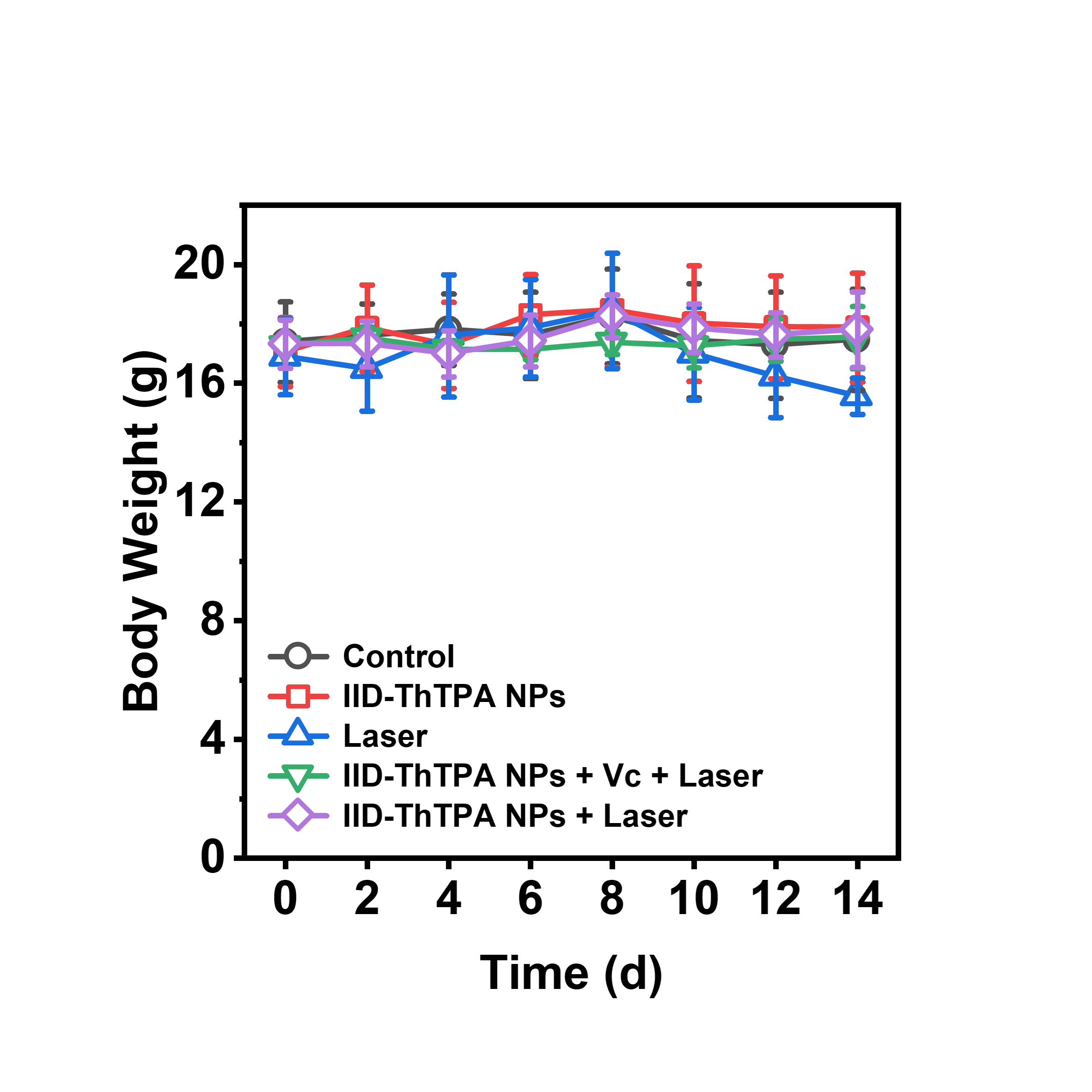


**Fig. S17** Body weight variations of the mice in different groups during the treatments (the results are presented as mean ± SD, n = 5)

**Table S1** Comparison of monocomponent-based single laser-excited materials for synergistic PTT/PDT reported in literature

| **Material** | **λ_ex._ (nm)** | **η (%)** | **Φ_Δ_ (%)** |
| --- | --- | --- | --- |
| DPP-TPA NPs [S6] | 660 | 34.5 | 33.6 |
| TBTPNPs [S4] | 635 | 37.1 | 40.0 |
| SeDPP-TPA NPs [S7] | 660 | 37.9 | 40.2 |
| FDPP-TPA NPs [S8] | 660 | 47.0 | 40.0 |
| di-BDP-NPs [S9] | 660 | 32.2 | 25.0 |
| tri-BDP-NPs [S9] | 785 | 45.2 | 18.0 |
| PDS-PDI [S10] | 660 | 40.0 | 16.7 |
| DPP-BDT NPs [S11] | 660 | 23.0 | 49.3 |
| P(DPP-BT/DOX) NPs [S12] | 730 | 50.0 | 27.3 |
| PDBr NPs [S13] | 660 | 35.7 | 67.0 |
| BDPTPA NPs [S14] | 808 | 52.6 | 35.2 |
| T-IPIC NPs [S15] | 808 | 39.6 | 2.30 |
| IID-ThTPA NPs | 671 | 35.4 | 84.0 |

**Table S2** Cartesian coordinates of the optimized geometries of IID-ThTPA and DPP-TPA

IID-ThTPA

Electronic Energy: -3948.126188 Hartree

| **Tag** | **Symbol** | **X** | **Y** | **Z** |
| --- | --- | --- | --- | --- |
| 1 | C | 2.483477 | -1.69997 | -0.28756 |
| 2 | C | 3.834176 | -1.98608 | -0.20674 |
| 3 | C | 4.746652 | -0.92557 | -0.01368 |
| 4 | C | 4.240423 | 0.383837 | 0.09225 |
| 5 | C | 2.878804 | 0.659518 | -0.0003 |
| 6 | C | 1.958292 | -0.38037 | -0.20258 |
| 7 | C | 0.498555 | -0.47803 | -0.29294 |
| 8 | C | 0.220084 | -1.96412 | -0.44686 |
| 9 | N | 1.443779 | -2.61023 | -0.46335 |
| 10 | C | -0.49855 | 0.478194 | -0.29295 |
| 11 | C | -0.22007 | 1.964298 | -0.44681 |
| 12 | N | -1.44377 | 2.610401 | -0.46332 |
| 13 | C | -2.48347 | 1.700131 | -0.28761 |
| 14 | C | -1.95828 | 0.380535 | -0.20266 |
| 15 | C | -3.83418 | 1.986233 | -0.20684 |
| 16 | C | -4.74666 | 0.925716 | -0.01387 |
| 17 | C | -4.24043 | -0.38369 | 0.09205 |
| 18 | C | -2.8788 | -0.65936 | -0.00045 |
| 19 | O | -0.84187 | -2.55805 | -0.58455 |
| 20 | C | 1.564693 | -4.05134 | -0.62302 |
| 21 | C | 1.667577 | -4.81085 | 0.707528 |
| 22 | O | 0.841887 | 2.558228 | -0.58445 |
| 23 | C | -1.56468 | 4.051514 | -0.62296 |
| 24 | C | -1.66761 | 4.811001 | 0.707594 |
| 25 | C | -6.17688 | 1.206437 | 0.078291 |
| 26 | C | 6.176863 | -1.20631 | 0.078539 |
| 27 | C | 6.804543 | -2.38728 | 0.420178 |
| 28 | C | 8.21837 | -2.30894 | 0.420964 |
| 29 | C | 8.709179 | -1.06597 | 0.075371 |
| 30 | S | 7.379866 | 0.023428 | -0.27051 |
| 31 | C | -6.8046 | 2.387456 | 0.419714 |
| 32 | C | -8.21842 | 2.309075 | 0.420486 |
| 33 | C | -8.7092 | 1.066029 | 0.075097 |
| 34 | S | -7.37984 | -0.02339 | -0.27057 |
| 35 | C | -10.102 | 0.633091 | -0.03275 |
| 36 | C | 10.102 | -0.63309 | -0.03254 |
| 37 | C | 11.12924 | -1.57114 | -0.25431 |
| 38 | C | 12.45782 | -1.18288 | -0.35539 |
| 39 | C | 12.82174 | 0.170651 | -0.23651 |
| 40 | C | 11.80447 | 1.115308 | -0.01587 |
| 41 | C | 10.47686 | 0.718944 | 0.079785 |
| 42 | N | 14.17536 | 0.570089 | -0.3409 |
| 43 | C | 14.51616 | 1.78091 | -1.00771 |
| 44 | C | 15.2054 | -0.24001 | 0.21474 |
| 45 | C | 15.48111 | 2.643837 | -0.46457 |
| 46 | C | 15.82174 | 3.821865 | -1.12682 |
| 47 | C | 15.19735 | 4.16833 | -2.3269 |
| 48 | C | 14.23129 | 3.315406 | -2.86502 |
| 49 | C | 13.89709 | 2.126378 | -2.21955 |
| 50 | C | 15.05512 | -0.80962 | 1.489187 |
| 51 | C | 16.06432 | -1.60712 | 2.025552 |
| 52 | C | 17.24367 | -1.83501 | 1.313169 |
| 53 | C | 17.39979 | -1.26095 | 0.049674 |
| 54 | C | 16.38869 | -0.4768 | -0.5026 |
| 55 | C | -11.1293 | 1.571099 | -0.25441 |
| 56 | C | -12.4579 | 1.182774 | -0.35544 |
| 57 | C | -12.8217 | -0.17078 | -0.23661 |
| 58 | C | -11.8044 | -1.11539 | -0.01608 |
| 59 | C | -10.4768 | -0.71896 | 0.07952 |
| 60 | N | -14.1753 | -0.57026 | -0.34092 |
| 61 | C | -14.5162 | -1.78116 | -1.00755 |
| 62 | C | -15.2054 | 0.239873 | 0.214714 |
| 63 | C | -15.0551 | 0.809371 | 1.489213 |
| 64 | C | -16.0643 | 1.606899 | 2.025564 |
| 65 | C | -17.2436 | 1.834914 | 1.313104 |
| 66 | C | -17.3997 | 1.260967 | 0.049552 |
| 67 | C | -16.3886 | 0.476796 | -0.50271 |
| 68 | C | -15.4812 | -2.64396 | -0.4643 |
| 69 | C | -15.8219 | -3.82206 | -1.12639 |
| 70 | C | -15.1975 | -4.16874 | -2.32641 |
| 71 | C | -14.2314 | -3.31594 | -2.86464 |
| 72 | C | -13.8971 | -2.12684 | -2.21934 |
| 73 | C | 1.808762 | -6.32366 | 0.499359 |
| 74 | C | 1.867049 | -7.11668 | 1.811371 |
| 75 | C | 2.010255 | -8.62982 | 1.603407 |
| 76 | C | 2.052451 | -9.41863 | 2.91614 |
| 77 | C | -1.80878 | 6.323815 | 0.49945 |
| 78 | C | -1.86706 | 7.116813 | 1.811478 |
| 79 | C | -2.01023 | 8.629957 | 1.603539 |
| 80 | C | -2.05239 | 9.418749 | 2.916287 |
| 81 | H | 4.192945 | -3.00383 | -0.31203 |
| 82 | H | 4.925623 | 1.207103 | 0.27107 |
| 83 | H | 2.530035 | 1.677541 | 0.073645 |
| 84 | H | -4.19293 | 3.003987 | -0.3121 |
| 85 | H | -4.92563 | -1.20697 | 0.270813 |
| 86 | H | -2.53004 | -1.67739 | 0.073478 |
| 87 | H | 0.674433 | -4.37522 | -1.16913 |
| 88 | H | 2.437606 | -4.25317 | -1.25532 |
| 89 | H | 0.768132 | -4.59647 | 1.29857 |
| 90 | H | 2.521493 | -4.43029 | 1.284188 |
| 91 | H | -0.6744 | 4.375407 | -1.16905 |
| 92 | H | -2.43757 | 4.253356 | -1.25529 |
| 93 | H | -0.76818 | 4.596609 | 1.298659 |
| 94 | H | -2.52154 | 4.430432 | 1.28422 |
| 95 | H | 6.260205 | -3.28049 | 0.70703 |
| 96 | H | 8.85995 | -3.13548 | 0.706534 |
| 97 | H | -6.2603 | 3.280728 | 0.706419 |
| 98 | H | -8.86002 | 3.135659 | 0.705899 |
| 99 | H | 10.8767 | -2.6191 | -0.38671 |
| 100 | H | 13.22414 | -1.92737 | -0.54474 |
| 101 | H | 12.06273 | 2.163582 | 0.092049 |
| 102 | H | 9.719048 | 1.471847 | 0.279611 |
| 103 | H | 15.95948 | 2.385009 | 0.474738 |
| 104 | H | 16.57077 | 4.478797 | -0.69241 |
| 105 | H | 15.46039 | 5.090928 | -2.83633 |
| 106 | H | 13.74221 | 3.567586 | -3.80228 |
| 107 | H | 13.15617 | 1.459689 | -2.64925 |
| 108 | H | 14.14676 | -0.62232 | 2.053131 |
| 109 | H | 15.93185 | -2.03982 | 3.013777 |
| 110 | H | 18.0312 | -2.45117 | 1.737506 |
| 111 | H | 18.30942 | -1.43431 | -0.51939 |
| 112 | H | 16.50885 | -0.04346 | -1.49051 |
| 113 | H | -10.8768 | 2.619077 | -0.38677 |
| 114 | H | -13.2242 | 1.92724 | -0.5447 |
| 115 | H | -12.0626 | -2.16369 | 0.091807 |
| 116 | H | -9.71894 | -1.47185 | 0.279262 |
| 117 | H | -14.1468 | 0.621969 | 2.053206 |
| 118 | H | -15.9319 | 2.039516 | 3.013831 |
| 119 | H | -18.0311 | 2.451099 | 1.737428 |
| 120 | H | -18.3092 | 1.434435 | -0.51957 |
| 121 | H | -16.5087 | 0.043521 | -1.49065 |
| 122 | H | -15.9596 | -2.38498 | 0.474958 |
| 123 | H | -16.5709 | -4.47889 | -0.69189 |
| 124 | H | -15.4606 | -5.09139 | -2.83572 |
| 125 | H | -13.7423 | -3.56828 | -3.80186 |
| 126 | H | -13.1562 | -1.46025 | -2.64912 |
| 127 | H | 0.964603 | -6.68665 | -0.10523 |
| 128 | H | 2.715043 | -6.52932 | -0.09009 |
| 129 | H | 0.957821 | -6.9155 | 2.396616 |
| 130 | H | 2.707216 | -6.75265 | 2.421417 |
| 131 | H | 1.174617 | -8.99016 | 0.986733 |
| 132 | H | 2.923202 | -8.83124 | 1.024997 |
| 133 | H | 2.898054 | -9.10526 | 3.540886 |
| 134 | H | 1.136414 | -9.26528 | 3.499758 |
| 135 | H | 2.154473 | -10.4944 | 2.733379 |
| 136 | H | -0.96461 | 6.686804 | -0.10512 |
| 137 | H | -2.71505 | 6.529502 | -0.09 |
| 138 | H | -0.95785 | 6.9156 | 2.396728 |
| 139 | H | -2.70724 | 6.752784 | 2.421506 |
| 140 | H | -1.17459 | 8.990291 | 0.986863 |
| 141 | H | -2.92318 | 8.831413 | 1.025142 |
| 142 | H | -2.89799 | 9.105395 | 3.541037 |
| 143 | H | -1.13635 | 9.265358 | 3.499892 |
| 144 | H | -2.15438 | 10.49453 | 2.733545 |

DPP-TPA

Electronic Energy: -3720.642125 Hartree

| **Tag** | **Symbol** | **X** | **Y** | **Z** |
| --- | --- | --- | --- | --- |
| 1 | C | -1.3229 | -1.14663 | -0.86943 |
| 2 | C | -0.70632 | 0.090654 | -0.7432 |
| 3 | C | 0.707434 | -0.08968 | -0.74259 |
| 4 | C | 0.986214 | -1.51309 | -0.824 |
| 5 | N | -0.31457 | -2.11801 | -0.87775 |
| 6 | C | -0.98506 | 1.514059 | -0.82503 |
| 7 | N | 0.315774 | 2.119003 | -0.87752 |
| 8 | C | 1.324113 | 1.147618 | -0.86835 |
| 9 | C | 2.725714 | 1.436612 | -1.05944 |
| 10 | C | -2.72444 | -1.43559 | -1.06121 |
| 11 | C | 3.326781 | 2.44461 | -1.79942 |
| 12 | C | 4.735798 | 2.378958 | -1.80555 |
| 13 | C | 5.246988 | 1.323951 | -1.06974 |
| 14 | S | 3.950501 | 0.389012 | -0.36772 |
| 15 | C | -3.32531 | -2.44321 | -1.80183 |
| 16 | C | -4.73435 | -2.37769 | -1.8081 |
| 17 | C | -5.24572 | -1.32317 | -1.07174 |
| 18 | S | -3.9494 | -0.38851 | -0.36905 |
| 19 | C | 6.649208 | 0.96461 | -0.85788 |
| 20 | C | -6.648 | -0.96416 | -0.85964 |
| 21 | C | -7.66145 | -1.93624 | -0.9669 |
| 22 | C | -8.99891 | -1.61243 | -0.78547 |
| 23 | C | -9.3825 | -0.29792 | -0.46321 |
| 24 | C | -8.37655 | 0.6768 | -0.34028 |
| 25 | C | -7.04259 | 0.349927 | -0.54468 |
| 26 | N | -10.7438 | 0.034119 | -0.26847 |
| 27 | C | -11.2468 | 1.288414 | -0.71774 |
| 28 | C | -11.622 | -0.88341 | 0.374773 |
| 29 | C | -12.1061 | 2.042046 | 0.097009 |
| 30 | C | -12.6071 | 3.262802 | -0.35164 |
| 31 | C | -12.248 | 3.759758 | -1.60627 |
| 32 | C | -11.3868 | 3.015358 | -2.41527 |
| 33 | C | -10.8955 | 1.784953 | -1.98291 |
| 34 | C | -11.2144 | -1.5713 | 1.528831 |
| 35 | C | -12.0779 | -2.47033 | 2.151981 |
| 36 | C | -13.3633 | -2.68463 | 1.649812 |
| 37 | C | -13.7745 | -1.99395 | 0.507866 |
| 38 | C | -12.9116 | -1.10666 | -0.13283 |
| 39 | C | 7.662877 | 1.936458 | -0.96521 |
| 40 | C | 9.000308 | 1.612286 | -0.78418 |
| 41 | C | 9.383667 | 0.297633 | -0.46226 |
| 42 | C | 8.377499 | -0.67685 | -0.3392 |
| 43 | C | 7.043567 | -0.34962 | -0.5432 |
| 44 | N | 10.74488 | -0.0348 | -0.26799 |
| 45 | C | 11.6237 | 0.882523 | 0.374768 |
| 46 | C | 11.24745 | -1.28924 | -0.71746 |
| 47 | C | 11.21684 | 1.570604 | 1.528971 |
| 48 | C | 12.08088 | 2.469432 | 2.151649 |
| 49 | C | 13.36614 | 2.683338 | 1.648859 |
| 50 | C | 13.77655 | 1.992457 | 0.506772 |
| 51 | C | 12.91315 | 1.10537 | -0.13346 |
| 52 | C | 12.10668 | -2.04317 | 0.097032 |
| 53 | C | 12.6072 | -3.26406 | -0.35179 |
| 54 | C | 12.2476 | -3.76084 | -1.60637 |
| 55 | C | 11.38643 | -3.01614 | -2.41512 |
| 56 | C | 10.89567 | -1.7856 | -1.98258 |
| 57 | O | 2.021597 | -2.16832 | -0.81283 |
| 58 | O | -2.02048 | 2.169238 | -0.81507 |
| 59 | C | 0.427535 | 3.566432 | -0.68588 |
| 60 | C | 0.059152 | 4.052658 | 0.737043 |
| 61 | C | -0.0146 | 5.594438 | 0.694205 |
| 62 | C | -0.46818 | 6.288172 | 1.985689 |
| 63 | C | -0.62758 | 7.805946 | 1.82064 |
| 64 | C | -1.0602 | 8.511602 | 3.109744 |
| 65 | C | -0.42639 | -3.56549 | -0.68671 |
| 66 | C | -0.06096 | -4.05203 | 0.73687 |
| 67 | C | 0.013393 | -5.59378 | 0.693684 |
| 68 | C | 0.463604 | -6.28794 | 1.986114 |
| 69 | C | 0.623758 | -7.80561 | 1.820868 |
| 70 | C | 1.052857 | -8.51173 | 3.110897 |
| 71 | C | -1.04545 | -3.50567 | 1.800484 |
| 72 | C | -0.39973 | -3.15043 | 3.146834 |
| 73 | C | 1.041199 | 3.505598 | 1.802572 |
| 74 | C | 0.392505 | 3.149982 | 3.147393 |
| 75 | H | 2.766728 | 3.171528 | -2.37524 |
| 76 | H | 5.360435 | 3.062749 | -2.36957 |
| 77 | H | -2.76512 | -3.16977 | -2.37798 |
| 78 | H | -5.35887 | -3.06122 | -2.37256 |
| 79 | H | -7.39552 | -2.96531 | -1.18987 |
| 80 | H | -9.75759 | -2.38162 | -0.88489 |
| 81 | H | -8.6478 | 1.697175 | -0.09074 |
| 82 | H | -6.2933 | 1.132817 | -0.46404 |
| 83 | H | -12.3763 | 1.665573 | 1.078531 |
| 84 | H | -13.2712 | 3.834252 | 0.29159 |
| 85 | H | -12.6344 | 4.715177 | -1.94941 |
| 86 | H | -11.1044 | 3.385861 | -3.39721 |
| 87 | H | -10.238 | 1.202738 | -2.62078 |
| 88 | H | -10.222 | -1.39543 | 1.93185 |
| 89 | H | -11.7469 | -2.99411 | 3.044944 |
| 90 | H | -14.0363 | -3.38046 | 2.142678 |
| 91 | H | -14.7697 | -2.15517 | 0.101905 |
| 92 | H | -13.2306 | -0.58136 | -1.02753 |
| 93 | H | 7.397173 | 2.965633 | -1.18796 |
| 94 | H | 9.759142 | 2.381314 | -0.88368 |
| 95 | H | 8.648552 | -1.69734 | -0.08989 |
| 96 | H | 6.294122 | -1.13235 | -0.46249 |
| 97 | H | 10.22458 | 1.395047 | 1.932464 |
| 98 | H | 11.75045 | 2.993372 | 3.04473 |
| 99 | H | 14.0395 | 3.379018 | 2.141359 |
| 100 | H | 14.77165 | 2.153365 | 0.100331 |
| 101 | H | 13.23152 | 0.579916 | -1.02827 |
| 102 | H | 12.37727 | -1.66682 | 1.078504 |
| 103 | H | 13.27124 | -3.83575 | 0.291239 |
| 104 | H | 12.63368 | -4.71637 | -1.94965 |
| 105 | H | 11.10375 | -3.38651 | -3.39701 |
| 106 | H | 10.23817 | -1.20315 | -2.62026 |
| 107 | H | 1.447668 | 3.869683 | -0.92078 |
| 108 | H | -0.24352 | 4.034724 | -1.41263 |
| 109 | H | -0.94679 | 3.670752 | 0.950932 |
| 110 | H | 0.972937 | 5.990473 | 0.407746 |
| 111 | H | -0.70334 | 5.884816 | -0.11267 |
| 112 | H | 0.252459 | 6.093388 | 2.791113 |
| 113 | H | -1.42477 | 5.85796 | 2.316503 |
| 114 | H | 0.323056 | 8.233949 | 1.470996 |
| 115 | H | -1.36134 | 8.008378 | 1.027593 |
| 116 | H | -2.02405 | 8.128735 | 3.467415 |
| 117 | H | -0.32675 | 8.358323 | 3.911091 |
| 118 | H | -1.1669 | 9.591955 | 2.958602 |
| 119 | H | -1.44586 | -3.86905 | -0.92407 |
| 120 | H | 0.246499 | -4.03328 | -1.41207 |
| 121 | H | 0.94439 | -3.66983 | 0.953077 |
| 122 | H | -0.97325 | -5.98997 | 0.404393 |
| 123 | H | 0.704397 | -5.88364 | -0.11144 |
| 124 | H | -0.25927 | -6.09363 | 2.789645 |
| 125 | H | 1.419201 | -5.85768 | 2.319741 |
| 126 | H | -0.3258 | -8.23363 | 1.468343 |
| 127 | H | 1.359805 | -8.00758 | 1.029823 |
| 128 | H | 2.015586 | -8.12879 | 3.471483 |
| 129 | H | 0.317084 | -8.35897 | 3.91021 |
| 130 | H | 1.160234 | -9.59199 | 2.959592 |
| 131 | H | -1.53154 | -2.60049 | 1.417511 |
| 132 | H | -1.85559 | -4.23318 | 1.954314 |
| 133 | H | 0.086928 | -4.01205 | 3.614872 |
| 134 | H | 0.36218 | -2.37238 | 3.017281 |
| 135 | H | -1.14851 | -2.76792 | 3.850409 |
| 136 | H | 1.527829 | 2.600421 | 1.420253 |
| 137 | H | 1.851213 | 4.232814 | 1.958412 |
| 138 | H | -0.09469 | 4.011599 | 3.614875 |
| 139 | H | -0.36953 | 2.372389 | 3.015844 |
| 140 | H | 1.13964 | 2.76673 | 3.852323 |

**S3 Supplementary References**

[S1] Gaussian 16, Revision A. 03, M. J. Frisch, G. W. Trucks, H. B. Schlegel, G. E. Scuseria, M. A. Robb et al., Gaussian, Inc., Wallingford CT. (2016).

[S2] S. Huang, R. K. Kannadorai, Y. Chen, Q. Liu, M. Wang, A narrow-bandgap benzobisthiadiazole derivative with high near-infrared photothermal conversion efficiency and robust photostability for cancer therapy. Chem. Commun. **51**, 4223-4226 (2015). <https://doi.org/10.1039/C4CC09399B>

[S3] D. K. Roper, W. Ahn, M. Hoepfner, Microscale heat transfer transduced by surface plasmon resonant gold nanoparticles. J. Phys. Chem. C **111**, 3636-3641 (2007). <https://doi.org/10.1021/jp064341w>

[S4] L. Guo, G. Niu, X. Zheng, J. Ge, W. Liu et al., Single near-infrared emissive polymer nanoparticles as versatile phototheranostics. Adv. Sci. **4**, 1700085 (2017). <https://doi.org/10.1002/advs.201700085>

[S5] R. W. Redmond, J. N. Gamlin, A compilation of singlet oxygen yields from biologically relevant molecules. Photochem. Photobiol. **70**, 391-475 (1999). <https://doi.org/10.1111/j.1751-1097.1999.tb08240.x>

[S6] Y. Cai, P. Liang, Q. Tang, X. Yang, W. Si, W. Huang, Q. Zhang, X. Dong, Diketopyrrolopyrrole-triphenylamine organic nanoparticles as multifunctional reagents for photoacoustic imaging-guided photodynamic/photothermal synergistic tumor therapy. ACS Nano **11**, 1054-1063 (2017). <https://doi.org/10.1021/acsnano.6b07927>

[S7] Y. Cai, P. Liang, W. Si, B. Zhao, J. Shao et al., A selenophene substituted diketopyrrolopyrrole nanotheranostic agent for highly efficient photoacoustic/infrared-thermal imaging-guided phototherapy. Org. Chem. Front. **5**, 98-105 (2018). <https://doi.org/10.1039/C7QO00755H>

[S8] P. Liang, Y. Wang, P. Wang, J. Zou, H. Xu et al., Triphenylamine flanked furan-diketopyrrolopyrrole for multi-imaging guided photothermal/photodynamic cancer therapy. Nanoscale **9**, 18890-18896 (2017). <https://doi.org/10.1039/C7NR07204J>

[S9] S. Ye, J. Rao, S. Qiu, J. Zhao, H. He et al., Rational design of conjugated photosensitizers with controllable photoconversion for dually cooperative phototherapy. Adv. Mater. **30**, 1801216 (2018). <https://doi.org/10.1002/adma.201801216>

[S10] P. Sun, X. Wang, G. Wang, W. Deng, Q. Shen et al., A perylene diimide zwitterionic polymer for photoacoustic imaging guided photothermal/photodynamic synergistic therapy with single near-infrared irradiation. J. Mater. Chem. B **6**, 3395-3403 (2018). <https://doi.org/10.1039/C8TB00845K>

[S11] Q. Wang, B. Xia, J. Xu, X. Niu, J. Cai et al., Biocompatible small organic molecule phototheranostics for NIR-II fluorescence/photoacoustic imaging and simultaneous photodynamic/photothermal combination therapy. Mater. Chem. Front. **3**, 650-655 (2019). <https://doi.org/10.1039/C9QM00036D>

[S12] Q. Wang, Y. Dai, J. Xu, J. Cai, X. Niu et al., All-in-one phototheranostics: single laser triggers NIR-II fluorescence/photoacoustic imaging guided photothermal/photodynamic/chemo combination therapy. Adv. Funct. Mater. **29**, 1901480 (2019). <https://doi.org/10.1002/adfm.201901480>

[S13] X. Yang, Q. Yu, N. Yang, L. Xue, J. Shao, B. Li, J. Shao, X. Dong, Thieno[3,2-b]thiophene-DPP based near-infrared nanotheranostic agent for dual imaging-guided photothermal/photodynamic synergistic therapy. J. Mater. Chem. B **7**, 2454-2462 (2019). <https://doi.org/10.1039/C8TB03185A>

[S14] J. Zhu, J. Zou, Z. Zhang, J. Zhang, Y. Sun, X. Dong, Q. Zhang, An NIR triphenylamine grafted BODIPY derivative with high photothermal conversion efficiency and singlet oxygen generation for imaging guided phototherapy. Mater. Chem. Front. **3**, 1523-1531 (2019). <https://doi.org/10.1039/C9QM00044E>

[S15] Q. Wang, J. Xu, R. Geng, J. Cai, J. Li et al., High performance one-for-all phototheranostics: NIR-II fluorescence imaging guided mitochondria-targeting phototherapy with a single-dose injection and 808 nm laser irradiation. Biomaterials **231**, 119671 (2020). <https://doi.org/10.1016/j.biomaterials.2019.119671>
